# Multifunctional nanoplatforms for tumor microenvironment remodeling: Toward precision and intelligent cancer therapy

**DOI:** 10.1016/j.mtbio.2025.102385

**Published:** 2025-10-09

**Authors:** Xiangying Deng, Xinglong Liu, Lin Zhao

**Affiliations:** a Department of Pathology, The Second Xiangya Hospital, Central South University, Changsha, Hunan, 41001l, China; b Hunan Clinical Medical Research Center for Cancer Pathogenic Genes Testing and Diagnosis, Changsha, Hunan, 410011, China; c Institute of Medical Sciences, National Clinical Research Center for Geriatric Disorders, Xiangya Hospital, Central South University, Changsha, Hunan, 410008, China

**Keywords:** Tumor microenvironment, Nanomedicine, Stimuli-responsive delivery, Immunotherapy, AI-therapeutic ecosystem

## Abstract

Tumor heterogeneity and therapeutic resistance remain formidable obstacles to effective cancer treatment. Remodeling the tumor microenvironment (TME)—which is characterized by hypoxia, acidosis, immunosuppression, and extracellular matrix (ECM) remodeling—has emerged as a promising strategy to overcome these barriers. In recent years, nanotechnology has enabled the development of multifunctional and stimuli-responsive platforms for targeted TME modulation. Inorganic, organic, and hybrid nanocarriers leverage enhanced permeability and retention (EPR) effects, surface ligand engineering, and responsive elements to achieve spatiotemporally controlled drug and gene delivery. These advanced nanoplatforms can simultaneously normalize tumor vasculature, reverse hypoxia, modulate immune suppression, and degrade the ECM, thereby sensitizing tumors to conventional and emerging therapies. Integrating these TME-oriented strategies with photothermal and photodynamic therapy, immunotherapy, and metabolic reprogramming allows the construction of comprehensive, multimodal treatment systems. Furthermore, the convergence of intelligent nanomaterials and artificial intelligence (AI)-guided precision delivery is fostering the concept of therapeutic ecosystems, which enable dynamic treatment feedback and personalized tumor management. This review systematically summarizes the recent progress in nanotechnology-driven TME remodeling, highlights representative mechanisms and design strategies for multifunctional nanoplatforms, and discusses future opportunities and challenges in the development of intelligent, patient-centric cancer therapy systems.

## Graphical abstract

**Figure:**
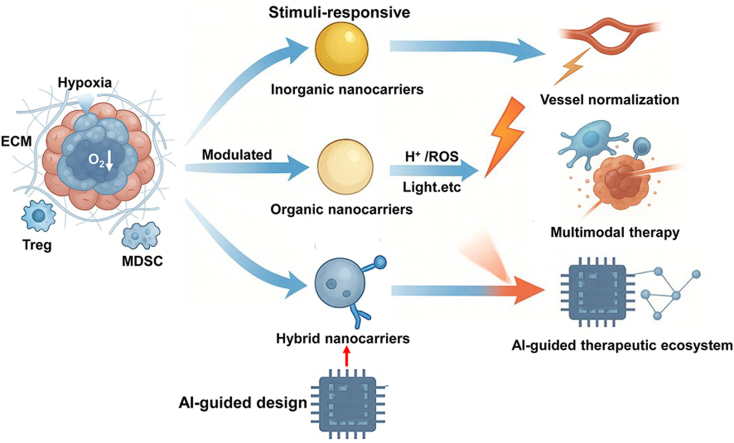

## 1 Introduction

Cancer has become one of the most formidable global health challenges, with the World Health Organization predicting that the number of new cases will exceed 22 million annually by 2030 [1]. The high heterogeneity and complexity of tumors hinder the establishment of universal treatment strategies, and therapeutic efficacy remains unsatisfactory, particularly in advanced and metastatic stages [2,3]. Conventional modalities such as surgery, chemotherapy, and radiotherapy remain the cornerstones of cancer treatment. However, each presents significant limitations. Surgery is restricted to localized lesions and often fails to eradicate micrometastases [4]. Chemotherapy and radiotherapy lack specificity and frequently cause severe systemic toxicity and immunosuppression [5]. In addition, their efficacy is further undermined by drug resistance and high recurrence rates [6,7].

In recent years, the TME has been recognized as a critical regulator of tumor progression and therapeutic resistance [8,9]. Hypoxia, immunosuppressive cells (such as tumor-associated macrophages, TAMs), and abnormal angiogenesis not only promote tumor growth but also diminish the efficacy of conventional therapies [[10], [11], [12], [13]]. Therefore, targeting and modulating the TME has emerged as an essential strategy to enhance therapeutic outcomes.

Against this backdrop, nanotechnology has demonstrated unique advantages. By virtue of tunable size, surface chemistry, and functional modifications, nanocarriers can selectively accumulate at tumor sites, enabling precise drug delivery while reducing off-target toxicity [[14], [15], [16], [17]]. Moreover, multifunctional nanoplatforms can co-deliver drugs, nucleic acids, or immune modulators to achieve synergistic therapeutic effects. Beyond drug delivery, nanomaterials also play a pivotal role in diagnosis and theranostics. For instance, quantum dots, gold nanoparticles, and magnetic nanoparticles can enhance imaging sensitivity and enable real-time monitoring [[18], [19], [20]]. In addition, stimuli-responsive or TME-modulating nanoplatforms can release therapeutics specifically under acidic or hypoxic conditions, or overcome traditional therapeutic barriers by promoting vascular normalization and immune reprogramming [[21], [22], [23], [24], [25]].

This review highlights nanointervention strategies for TME remodeling, summarizing their mechanisms, therapeutic potential, and translational challenges. It further introduces the concept of a nanotechnology-enabled therapeutic ecosystem, which integrates targeted delivery, diagnostics, immune modulation, and microenvironmental regulation to advance precision oncology.

## 2 Basic features of the TME and therapeutic targets

### 2.1 Composition and function of the TME

The TME is not solely composed of malignant tumor cells but also includes a variety of non-malignant components, such as stromal fibroblasts, endothelial cells, pericytes, and multiple immune cell types (e.g., macrophages, T cells, B cells, dendritic cells, and NK cells) [9,26]. These non-malignant cells engage in complex interactions with tumor cells through cytokine secretion, ECM remodeling, and metabolic crosstalk, collectively shaping tumor initiation, progression, therapeutic resistance, and immune evasion. Among them, TAMs are often polarized toward the M2 phenotype, secreting anti-inflammatory cytokines (e.g., IL-10, TGF-β) and various growth factors that promote tumor proliferation, angiogenesis, and immunosuppression [27]. In contrast, T cells and NK cells, which are inherently cytotoxic under physiological conditions, become functionally impaired within the TME due to immunosuppressive signals such as the PD-L1/PD-1 axis, leading to diminished antitumor activity [28,29]. Cancer-associated fibroblasts (CAFs) contribute to tumor progression by secreting cytokines and remodeling the ECM, which not only facilitates tumor cell migration and invasion but also affects drug delivery and therapeutic responsiveness [30,31].

In addition, vascular endothelial cells and lymphatic networks within the TME provide essential structural support for tumor growth and metastasis [9]. Tumor-associated vasculature often exhibits structural disorganization and functional defects, leading to local hypoxia and acidosis that not only enhance tumor aggressiveness but also promote therapeutic resistance [32]. At the same time, the lymphatic system serves as a conduit for tumor cell dissemination [9]. As the structural scaffold of the TME, the ECM—composed of proteins such as collagen and fibronectin, as well as polysaccharides—profoundly influences tumor cell behavior and drug penetration through its stiffness and density [33]. Excessive ECM deposition and cross-linking not only exacerbate tumor invasiveness but also severely hinder the delivery of anticancer drugs [34]. Importantly, ECM dysregulation is not merely a physical barrier but also a critical therapeutic target: degradation or remodeling of key components such as collagen, hyaluronic acid, and fibronectin has been shown to improve vascular perfusion, enhance intratumoral drug distribution, and reduce tissue stiffness, thereby markedly increasing sensitivity to subsequent therapies [35,36]. For example, a study developed smart nanogels loaded with hyaluronidase (HAase) and engineered them onto the surface of CAR-T cells, enabling precise HAase release under high ROS conditions in the TME [37]. This strategy effectively degraded the ECM, significantly promoted CAR-T cell infiltration, and enhanced their antitumor activity against solid tumors [37]. In summary, excessive ECM deposition and fibrosis are not only key drivers of tumor progression but also critical therapeutic targets, laying the foundation for ECM-targeted nanotechnology strategies.

Overall, the dynamic interplay between tumor cells and non-malignant components such as vasculature, lymphatics, and the ECM establishes a sophisticated regulatory network that not only sustains tumor progression but also profoundly influences therapeutic responses.

### 2.2 Dynamic properties of the TME

#### 2.2.1 Metabolic dysregulation

The TME is highly dynamic, with metabolic dysregulation representing one of its key hallmarks [12]. Rapid proliferation of tumor cells leads to increased local oxygen consumption, resulting in hypoxia. In response, tumor cells secrete hypoxia-inducible factors (HIFs), which promote angiogenesis and metabolic reprogramming [38,39]. Meanwhile, tumor cells preferentially rely on anaerobic glycolysis, leading to lactate accumulation and the establishment of an acidic microenvironment [40]. This acidic milieu not only enhances tumor cell invasiveness but also suppresses immune cell function. Recent studies have demonstrated that lactate accumulation directly diminishes the cytotoxicity of CD8^+^ T cells and reduces IFN-γ secretion, thereby weakening antitumor immunity [41,42]. At the same time, competition for glucose and glutamine between tumor and stromal cells and infiltrating lymphocytes further limits immune cell activity, whereas blockade of glutamine metabolism has been reported to restore T-cell effector function and enhance tumor control [43]. In addition, hypoxia-induced stabilization of HIF-1α drives the reprogramming of tumor-associated macrophages toward an immunosuppressive M2-like phenotype, thereby further promoting immune evasion and tumor progression [11]. Collectively, these findings highlight that metabolic dysregulation profoundly reshapes the TME, fostering tumor survival, immune escape, and therapeutic resistance.

#### 2.2.2 Immune evasion

Immune evasion is another prominent feature of the TME [44]. Tumors weaken anti-tumor immune responses by recruiting and activating immunosuppressive cells, such as regulatory T cells (Tregs) and myeloid-derived suppressor cells (MDSCs). Tregs suppress effector T cell function through the secretion of immunosuppressive cytokines, including IL-10 and TGF-β [45,46], while MDSCs inhibit T cell activity by releasing reactive oxygen species (ROS) and nitrogen metabolites [47]. Evidence indicates that tumor-derived chemokines promote the accumulation of Tregs within the TME, which suppresses CD8^+^ T cell cytotoxicity and is associated with poor patient prognosis [48,49]. In addition, tumor cells can express immune checkpoint molecules such as PD-L1, which directly impair T cell cytotoxicity by engaging with T cell receptors [50,51]. This intricate immunosuppressive network enables tumors to effectively evade immune surveillance, thereby accelerating tumor progression and metastasis.

#### 2.2.3 Abnormal angiogenesis

Angiogenesis plays a critical role in the TME; however, neovasculature often exhibits structural abnormalities and functional deficiencies. Tumor vessels are typically disorganized, tortuous, and highly permeable, leading to heterogeneous distribution of oxygen and nutrients within tumor tissues. Although angiogenesis supports tumor cell proliferation and dissemination, these structural abnormalities exacerbate hypoxia and severely hinder drug delivery. Studies in colorectal cancer have demonstrated that anti-VEGF (vascular endothelial growth factor) therapy (e.g., bevacizumab) can transiently “normalize” the aberrant vascular network by pruning immature vessels and reinforcing the remaining ones, thereby improving perfusion, alleviating hypoxia, and significantly enhancing the delivery and efficacy of concomitant chemotherapeutic agents [52,53]. Nonetheless, in most cases, dysfunctional vasculature continues to restrict the effective penetration of chemotherapeutic drugs and immune cells into the tumor core, ultimately compromising overall therapeutic outcomes [54,55]. Therefore, vascular normalization and functional restoration have emerged as key strategies to improve anti-tumor therapy.

#### 2.2.4 Chronic inflammation

In addition, chronic inflammation is persistently present within the TME and exerts a “double-edged sword” effect during tumor initiation and progression. On the one hand, inflammatory cells secrete a variety of cytokines and chemokines, such as interleukin-6 (IL-6) and tumor necrosis factor-α (TNF-α), which promote tumor cell proliferation, angiogenesis, and ECM remodeling. Study has shown that sustained IL-6-mediated activation of the JAK/STAT3 signaling pathway not only promotes the autonomous proliferation of cancer cells but also contributes to the development of a fibrotic and highly immunosuppressive TME [56,57]. On the other hand, certain inflammatory mediators have the potential to stimulate anti-tumor immunity; however, within the tumor context, inflammation is frequently skewed toward a pro-tumor phenotype [57,58]. For instance, tumor-associated macrophages and neutrophils enhance tumor invasiveness and reinforce immunosuppression through the release of pro-inflammatory mediators [59]. Therefore, the sustained presence of chronic inflammation not only fuels tumorigenesis and progression but also represents a critical barrier to effective cancer therapy.

**Fig. 1:**
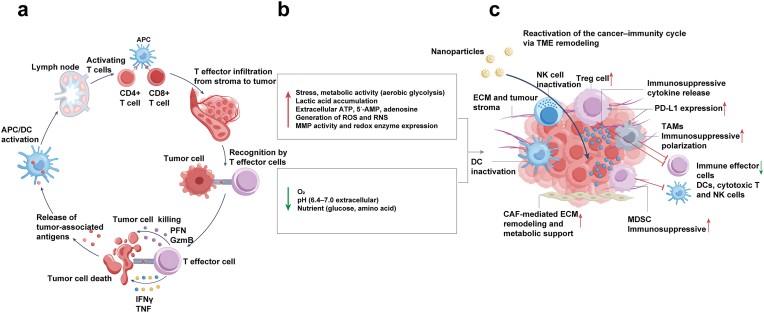
Key TME immunosuppressive features affecting anti-tumor immunity. (a) A functional cancer–immunity cycle activates T cells in draining lymph nodes, enabling tumor cell killing. (b) The TME, enriched with diverse surface markers and cytokines, suppresses immune elimination of tumors. (c) Tumor-responsive nanoparticles release therapeutic agents selectively within the TME, restoring the cancer–immunity cycle. The color scheme of this figure was partially adapted from references [[214], [215], [216], [217]].

In summary, TME-associated metabolic, immune, vascular, and inflammatory abnormalities collectively foster tumor progression and resistance, underscoring the need for comprehensive interventions (Fig. 1a–c).

### 2.3 Key therapeutic targets in the TME

Normalization of the tumor vasculature represents a critical strategy for improving drug delivery and therapeutic efficacy. The tumor neovasculature typically shows abnormal architecture. It is highly permeable, disorganized, and functionally deficient. These abnormalities cause hypoxia and acidosis in tumor tissues and restrict the infiltration of drugs and immune cells. Targeting angiogenic signaling pathways, such as the VEGF pathway, can partially restore vascular structure and function, thereby increasing the supply of oxygen and nutrients and alleviating hypoxia. For example, anti-VEGF agents such as bevacizumab have been shown to inhibit excessive angiogenesis and improve vascular permeability, consequently enhancing the intratumoral distribution of chemotherapeutic agents and immune cells [26,60]. Moreover, the combined use of nanodrug delivery systems with vascular modulators can further optimize drug delivery efficiency, offering novel avenues for tumor therapy.

Modulating the functional balance of immune cells is another key therapeutic direction. Immunosuppressive cells within the TME, such as Tregs and MDSCs, suppress the antitumor activities of effector T cells and NK cells [61]. Immune checkpoint blockade, which involves targeting molecules such as programmed death-1 (PD-1)/PD-L1 and cytotoxic T-lymphocyte-associated antigen 4 (CTLA-4), can restore T cell function and reactivate anti-tumor immune responses [62,63]. In addition, reprogramming TAMs from the pro-tumoral M2 phenotype to the anti-tumoral M1 phenotype represents an effective strategy to restore antitumor immunity and enhance local immune responses. Furthermore, targeting chemokine and cytokine signaling to limit the recruitment and activation of immunosuppressive cells can synergistically disrupt immune evasion mechanisms within the tumor.

Modifying the metabolic landscape of tumors to enhance anti-tumor immunity is also a critical therapeutic target. Metabolic reprogramming in tumor cells—such as the shift toward aerobic glycolysis—results in an acidic microenvironment and nutrient depletion, which together impair immune cell function. Inhibiting key metabolic enzymes, such as lactate dehydrogenase (LDH), can reduce lactate accumulation and improve extracellular pH conditions [64]. Additionally, modulating glutamine metabolism or targeting lipid metabolic pathways can attenuate the metabolic advantage of tumor cells and create a more favorable metabolic niche for immune cells [65]. For example, the use of metabolic modulators or nanoparticle-based delivery systems can effectively reverse nutrient competition, thereby enhancing the anti-tumor activity of immune cells [66].

Overall, the TME imposes barriers such as hypoxia, acidosis, stromal density, angiogenic abnormalities, and immune suppression, which drive tumor progression and weaken conventional therapies, underscoring the need for innovative strategies.

Nanotechnology offers unique advantages in overcoming these obstacles. Through tunable size, charge, and surface modifications, nanoplatforms can penetrate physical barriers and preferentially accumulate at tumor sites. Their stimulus-responsive properties enable controlled drug release in response to TME-specific conditions such as pH, ROS, or enzymatic activity. Furthermore, multifunctional designs allow for vascular normalization to improve perfusion, degradation of excessive ECM to facilitate drug penetration, and immune cell reprogramming to restore antitumor activity. Collectively, these unique capabilities establish nanotechnology as an indispensable approach for surmounting TME-associated barriers and advancing precision cancer therapy.

## 3 Mechanism of nanotechnology in TME intervention

### 3.1 Classification and characterization of nanocarriers

Nanocarriers, as essential platforms for drug delivery, can be broadly classified into three categories based on their material composition: inorganic, organic, and hybrid nanocarriers [67] (Table 1).

**Table 1:** Nanocarriers of different material types.

Category	Material Composition	Key Features	Applications	Representative Examples	Refs
Inorganic	Metals (e.g., gold, silver), metal oxides (e.g., Fe_3_O_4_), silica	High structural stability, tunable particle size, easy surface modification, multimodal imaging	Image-guided drug delivery, photothermal/magnetic hyperthermia, theranostics	Gold nanoparticles, mesoporous silica (MSNs), magnetic nanoparticles	[[234], [235], [236]]
Organic	Polymers (e.g., PLGA, PCL), lipids (e.g., phospholipids), polysaccharides	Good biocompatibility, biodegradability, controlled release, modifiable for targeting ligands	Chemotherapy delivery, gene delivery (siRNA/mRNA), immune modulation	Liposomes, polymeric nanoparticles, micelles	[[237], [238], [239]]
Hybrid	Combination of organic and inorganic components (e.g., metal-polymer, lipid-silica)	Integrates advantages of both types, modular functional design, high drug loading, multimodal therapy	Targeted chemotherapy combined with photothermal/photodynamic/immunotherapy	Metal-organic frameworks (MOFs), core–shell nanoparticles	[[240], [241], [242]]

Inorganic nanocarriers are of particular importance in cancer therapy due to their high structural stability, unique physicochemical properties, and strong potential for functionalization [68]. For example, gold nanoparticles, with their excellent photothermal conversion efficiency, are widely used in photothermal therapy (PTT) and can also serve as imaging contrast agents, thereby enabling theranostic applications [69]. Iron oxide nanoparticles, owing to their superparamagnetic properties, are commonly employed in magnetic resonance imaging (MRI) and magnetically guided therapies [70]. Silica nanoparticles, with favorable biocompatibility and tunable porous structures, can efficiently load anticancer drugs or genetic molecules for controlled release and targeted delivery [71]. Nevertheless, their poor biodegradability poses a major concern, as they may accumulate in organs such as the liver and spleen, leading to potential chronic toxicity. Furthermore, large-scale synthesis of inorganic nanocarriers often struggles with uniform control over size and surface chemistry, typically requiring complex processes such as high-temperature reduction or template-assisted synthesis, which significantly hinders clinical translation.

Organic nanocarriers, characterized by superior biocompatibility and design flexibility, have become the mainstream in drug delivery. Liposomes, the first nanocarriers to reach clinical use, feature a phospholipid bilayer structure resembling biological membranes, capable of encapsulating both hydrophilic and hydrophobic drugs, with liposomal doxorubicin (Doxil) serving as a representative example [72]. Polymeric nanoparticles constitute another major class, offering stability and controlled drug release, and their targeting capacity can be greatly enhanced through polymer chain modification [73]. Polymeric micelles, formed via self-assembly with hydrophobic cores and hydrophilic shells, allow efficient loading of hydrophobic drugs, extend circulation time, and reduce systemic toxicity [74]. Compared with inorganic carriers, organic nanocarriers exhibit better biodegradability and in vivo safety, and some formulations (e.g., liposomes and polymeric nanomedicines) have already achieved scalable clinical production using methods such as thin-film hydration, ethanol injection, and microfluidics. However, their limitations include insufficient physical stability (e.g., premature drug leakage) and batch-to-batch variability during polymer synthesis, which remain challenges for industrial-scale translation.

Hybrid nanocarriers integrate the advantages of both inorganic and organic systems, thereby exhibiting multifunctional synergies [75]. For example, gold nanoparticle–liposome hybrids enable simultaneous photothermal therapy and drug delivery, while silica–polymer hybrids combine the structural stability of inorganic carriers with the biocompatibility of organic materials [76,77]. These composite systems often demonstrate higher drug-loading capacities and greater potential for functionalization, and with stimuli-responsive designs (e.g., pH, light, or magnetic field), they can achieve precise controlled release [78]. Their flexible architectures also allow the integration of imaging, diagnostic, and therapeutic functions, accelerating the development of unified nanotechnology-based cancer treatments [79]. However, hybrid nanocarriers face the greatest translational challenges: their synthesis is complex and costly, large-scale manufacturing struggles to maintain batch uniformity, and comprehensive quality control standards are lacking. Moreover, long-term biosafety data remain scarce, necessitating systematic animal studies and large-scale clinical trials.

In summary, inorganic, organic, and hybrid nanocarriers exhibit distinct strengths and limitations that shape their clinical translation potential. Inorganic carriers offer high stability, drug-loading capacity, and unique optical or magnetic properties but are hindered by poor biodegradability, potential toxicity, and complex synthesis. Organic carriers provide superior biocompatibility and biodegradability, with several formulations in clinical use, yet face challenges of instability, rapid clearance, and batch variability. Hybrid systems integrate the robustness of inorganic and the safety of organic carriers, enabling multifunctional and stimuli-responsive designs, but their synthesis complexity and high cost impede industrialization. Future efforts should emphasize long-term biosafety evaluation, scalable manufacturing standards, and AI-driven design to accelerate the clinical translation of nanomedicine.

### 3.2 Mechanisms of nano-intervention

Nanotechnology-based interventions are increasingly occupying a central role in cancer therapy, with advantages extending beyond precise delivery to include intelligent responsiveness and multifunctional synergy [15,80]. The introduction of nanotechnology has expanded therapeutic strategies from simple drug delivery to encompass multimodal diagnosis, therapy, and real-time monitoring. The following sections elaborate on three core mechanisms: targeted delivery, intelligent responsiveness, and multifunctional integration.

**Fig. 2:**
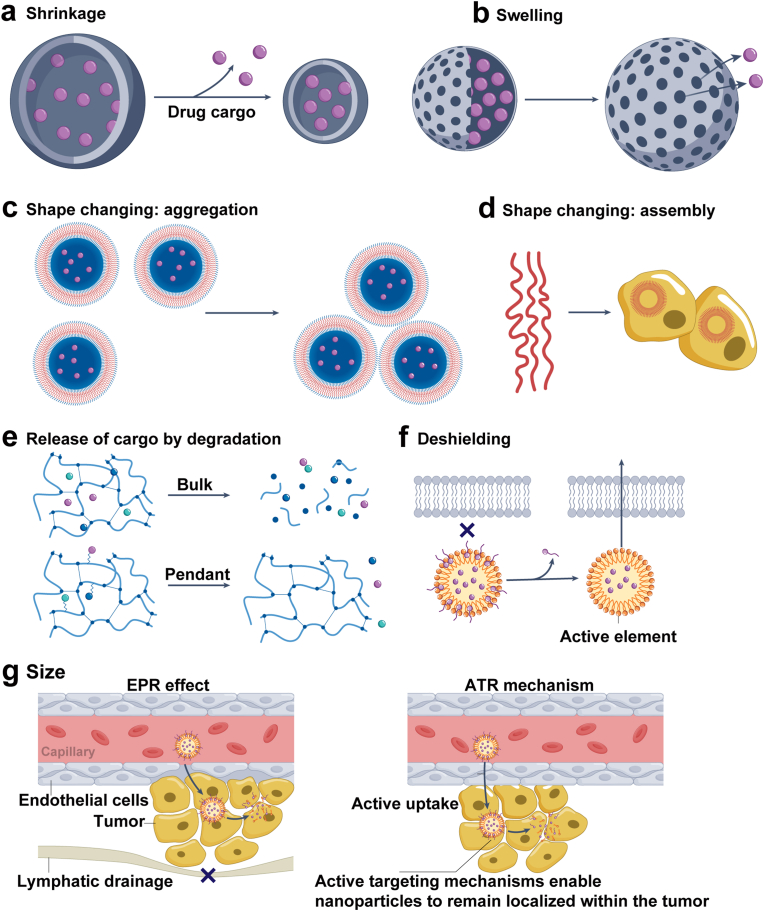
Stimuli-responsive release mechanisms in tumor-targeted nanomaterials. (a–b) Size modulation: responsive elements induce nanoparticle shrinkage or swelling to promote cargo release. (c–d) Tumor-triggered assembly transforms nanoparticles into larger aggregates or new structures, enhancing retention. (e) Controlled release occurs via backbone degradation or cleavage of tumor-sensitive linkers. (f) Stimuli-responsive deshielding removes protective layers, exposing bioactive components for higher specificity. (g) Size-dependent behavior: tumor uptake and retention are governed by EPR (passive accumulation) and ATR (active uptake and interactions). The color scheme of this figure was partially adapted from references [[218], [219], [220]].

Targeted delivery is one of the most prominent features of nanotechnology-enabled interventions. Compared with conventional delivery systems, nanomaterials can more precisely localize therapeutic agents to tumor sites, thereby improving efficacy and reducing off-target toxicity [81]. This mechanism often exploits pathological features of tumors, particularly the EPR effect [82] (Fig. 2a–g), which facilitates nanoparticle extravasation through abnormal vasculature and prolonged retention within the TME [83]. Importantly, the physicochemical properties of nanoparticles directly affect delivery outcomes: (i) Size: particles <10 nm is rapidly cleared renally, whereas those >200 nm is readily captured by the mononuclear phagocyte system; sizes between 50 and 150 nm are generally optimal for tumor accumulation via the EPR effect. (ii) Surface charge: positively charged particles enhance cellular uptake via electrostatic interactions but may increase serum protein adsorption and systemic toxicity; neutral or slightly negative charges prolong circulation. (iii) Shape: rod-, sheet-, or disk-like nanoparticles exhibit distinct hemodynamic profiles and extravasation efficiencies compared with spherical ones, thereby influencing tumor penetration and uptake. Furthermore, surface functionalization with ligands such as antibodies, peptides, or aptamers enables active targeting, substantially improving specificity and even overcoming biological barriers such as the blood-brain barrier (BBB), which is particularly advantageous for central nervous system tumors [84].

**Fig. 3:**
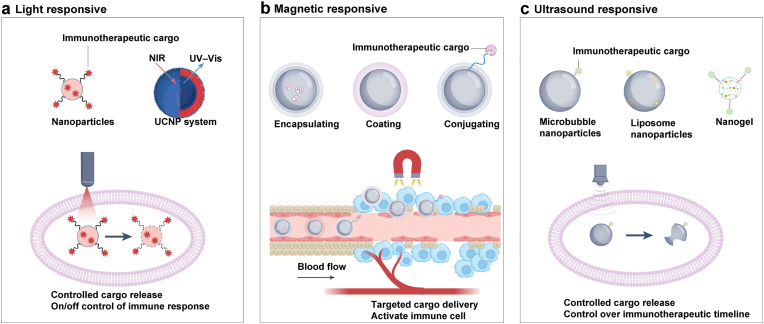
Nanomaterials combined with exogenous stimuli for enhancing cancer immunotherapy. (a) Light-responsive systems: Nanomaterials such as upconversion nanoparticles (UCNPs) use UV or NIR light to trigger immunotherapeutic release and regulate immune responses. (b) Magnetic-responsive systems: Immunotherapeutics are loaded or conjugated onto magnetic nanomaterials, enabling targeted delivery under an external magnetic field. (c) Ultrasound-responsive systems: Microbubbles, liposomes, and nanogels release drugs and enhance intracellular delivery upon ultrasound stimulation, allowing spatial control of immunotherapy. The color scheme of this figure was partially adapted from references [[221], [222], [223], [224], [225], [226], [227]].

Intelligent responsiveness is another critical property, enabling nanomaterials to undergo structural or functional changes in response to specific stimuli, thereby conferring higher precision and adaptability [85,86]. These triggers include endogenous TME features (acidic pH, elevated ROS, overexpressed enzymes) and exogenous stimuli (light, heat, magnetic fields, ultrasound) [87]. For example, pH-sensitive carriers disassemble in acidic milieus and release drugs. ROS-responsive systems exploit oxidative stress for controlled release or cytotoxic species generation. Thermosensitive nanoparticles release drugs under elevated temperatures during hyperthermia. Photosensitive particles alter conformation or generate heat upon irradiation for photothermal or photodynamic therapy [[88], [89], [90]]. Magnetic nanoparticles allow site-specific localization, controlled release, or hyperthermia under external magnetic fields [91]. Particle design also influences these responses. Smaller nanoparticles are more sensitive to pH or ROS changes because of their larger surface area, whereas anisotropic structures possess enhanced optical properties that enable efficient photothermal conversion. Collectively, these mechanisms ensure maximum therapeutic effect within the TME while minimizing damage to normal tissues (Fig. 3a–c).

Multifunctionality represents another hallmark of nanotechnology in oncology. By integrating diagnostic, therapeutic, and monitoring functions into a single nanoplatform, nanointerventions enable real-time feedback and dynamic treatment adjustments [92]. Nanomaterials can encapsulate fluorescent probes, radiotracers, or MRI contrast agents, thereby facilitating early tumor detection and continuous monitoring [93,94]. At the same time, they can act as carriers for chemotherapeutics, gene agents, or immunomodulators, enabling imaging-guided therapy [83]. Theragnostic nanoplatforms thus combine drug delivery with imaging modalities, allowing precise monitoring of biodistribution and therapeutic response in real time. Moreover, platforms integrating chemotherapy with hyperthermia or phototherapy exemplify multimodal synergy. Such strategies lay the groundwork for individualized and precision oncology.

In summary, nanotechnology offers transformative cancer therapies through targeted delivery, intelligent responsiveness, and multifunctional integration. Future progress hinges on coupling physicochemical properties with biological mechanisms to achieve effective clinical translation.

Therefore, nanocarriers can be understood from two complementary perspectives. From the material perspective, they are classified into inorganic, organic, and hybrid systems, whose intrinsic properties (e.g., particle size, surface charge, degradability, and stability) determine circulation, drug-loading efficiency, and biosafety. From the mechanistic perspective, their functions include targeted delivery, stimuli-responsive release, ECM remodeling, vascular normalization, and immune reprogramming. Material properties define what nanocarriers can achieve, while mechanisms explain how they exert their effects; together, they form the framework for nanoplatform design. In clinical translation, inorganic carriers possess strong stability but suffer from poor degradability and complex production; organic carriers exhibit biocompatibility and biodegradability, with some already in clinical use, though they face issues of poor stability and rapid clearance; hybrid carriers integrate these advantages but remain limited by complex synthesis, high cost, and insufficient long-term biosafety data. Mechanistic applications are also constrained by tumor heterogeneity, off-target effects, dose dependency, and immune-related risks. Future efforts should focus on developing low-toxicity biodegradable materials, establishing standardized large-scale manufacturing processes, and integrating AI with multi-omics to accelerate the clinical translation of nanomedicine.

### 3.3 Tumor microenvironment-specific regulatory strategies

The TME is a critical determinant of tumor growth, metastasis, and therapy resistance [9]. In recent years, nanotechnology-based strategies for TME modulation have garnered significant attention. These approaches enable precise intervention through mechanisms such as alleviating hypoxia, metabolic reprogramming, immune activation, and vascular normalization, thereby enhancing therapeutic efficacy while effectively reducing systemic toxicity [95]. Studies have demonstrated that Doxil combined with immune checkpoint inhibitors leads to a higher objective response rate (ORR) and prolonged median progression-free survival (PFS) in patients with solid tumors, showing markedly superior efficacy compared to conventional chemotherapy [96,97]. Meanwhile, TME-responsive nanoparticles have been shown to enhance intratumoral drug penetration efficiency by 2–3 times, reduce hypoxia levels by approximately 40 %, and promote T cell infiltration, collectively contributing to significantly improved treatment outcomes [[98], [99], [100]]. In summary, these findings underscore the quantifiable and translational value of nanotechnology in TME remodeling, providing strong support for precision cancer therapy.

**Fig. 4:**
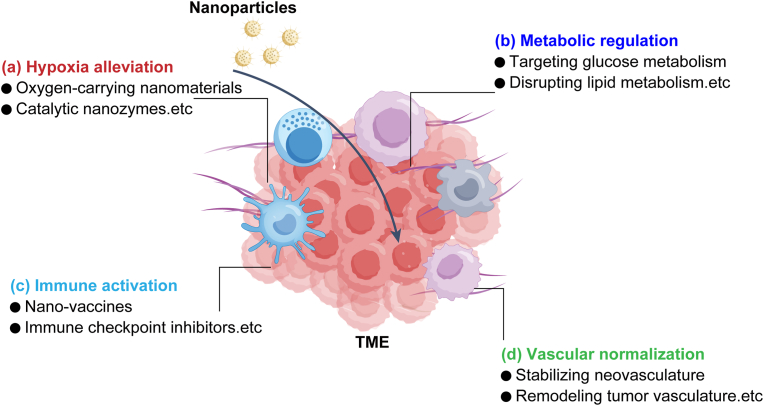
Nanotechnology-based strategies for TME modulation. Nanotechnology remodels the TME through four key mechanisms: (a) hypoxia alleviation, (b) metabolic regulation, (c) immune activation, and (d) vascular normalization, collectively enhancing therapeutic efficacy and enabling precision cancer therapy. The color scheme of this figure was partially adapted from references [[214], [215], [216], [217]].

Hypoxia is a common feature of the TME and is known to promote tumor cell proliferation, invasion, and resistance to treatment [38] (Fig. 4a). The application of oxygen-carrying nanomaterials and catalytic nanozymes offers novel approaches for alleviating hypoxia. Oxygen-carrying nanomaterials can load and release oxygen specifically at tumor sites, mitigating hypoxia-induced treatment resistance, particularly enhancing the efficacy of radiotherapy [101]. Furthermore, catalytic nanozymes—such as catalase—can decompose hydrogen peroxide (H_2_O_2_) in the TME to generate oxygen, thereby reducing oxidative stress in the tumor area and improving hypoxic conditions, ultimately enhancing therapeutic outcomes [102].

Tumor cells exhibit distinct metabolic profiles (Fig. 4b), including a preference for glycolysis over oxidative phosphorylation even under normoxic conditions—a phenomenon known as the Warburg effect [103]. This metabolic reprogramming leads to lactate accumulation and acidification of the TME. Nanotechnology can intervene in tumor metabolism by targeting glucose and lipid metabolism, as well as lactate accumulation. Specifically designed nanocarriers targeting tumor cells can inhibit glucose transport and enzyme activity, thereby reducing the energy supply to cancer cells [104]. Additionally, nanomaterials can disrupt lipid metabolism by limiting fatty acid uptake, thus restraining tumor growth [105,106]. Simultaneously, nanoparticles can facilitate lactate clearance or neutralize the acidic microenvironment, imposing metabolic stress on tumor cells and suppressing tumor progression [107].

Immune evasion is a major obstacle to effective antitumor immunity (Fig. 4c). Nanotechnology, using nanovaccines and immune checkpoint inhibitors, can effectively activate antitumor immune responses [108,109]. Nanovaccines deliver tumor antigens via nanocarrier systems, enhancing immune recognition and prolonging immune activation through sustained-release mechanisms [110]. Moreover, immune checkpoint inhibitors can be delivered specifically to tumor sites via nanocarriers, directly blocking tumor immune evasion mechanisms and restoring the immune system’s cytotoxic function [111]. These immune-regulating strategies leverage the precise delivery capability of nanotechnology to reduce systemic toxicity and significantly improve therapeutic efficacy.

Abnormal tumor angiogenesis is typically characterized by irregular and dysfunctional vascular structures, which lead to hypoxia and hinder drug delivery (Fig. 4d). Abnormal tumor angiogenesis is typically characterized by structural disorganization and functional deficiencies, leading to hypoxia and impaired drug delivery [54,112]. These vessels often exhibit excessive branching, irregular lumen size, and increased permeability, resulting in insufficient blood perfusion and inadequate oxygen supply within tumor regions. Moreover, vascular leakage contributes to elevated interstitial fluid pressure, which further restricts the effective penetration of therapeutic agents into the tumor core. To address these challenges, nanomaterials can promote vascular normalization by stabilizing neovasculature and optimizing vascular architecture, thereby improving drug transport and oxygen delivery [113]. Nanomaterials have been employed to promote vascular normalization by stabilizing neovasculature and optimizing vascular structure, thereby improving drug transport and oxygen delivery. Nanomaterials can modulate endothelial cell function, remodel tumor vasculature, and reduce vascular leakage, leading to improved drug distribution and enhanced therapeutic responses [114]. In addition to enhancing chemotherapeutic drug delivery, vascular normalization facilitated by nanotechnology can improve the infiltration of immune cells into tumor tissues, thereby increasing immunotherapy efficacy. Moreover, by stabilizing tumor microvessels, nanotechnology can help slow tumor growth and improve overall treatment outcomes.

In summary, nanotechnology-based TME-specific regulatory strategies can synergistically alleviate hypoxia, remodel metabolism, activate immunity, and promote vascular normalization, thereby not only enhancing drug penetration and antitumor efficacy but also providing solid support for precision and comprehensive cancer therapy.

## 4 Specific application directions of nanointerventions

### 4.1 TME-targeted drug and gene delivery via nanotechnology

With the extensive application of nanotechnology in the biomedical field, nanotechnology-mediated interventions have attracted increasing attention in the areas of drug delivery and gene therapy. Designing nanocarriers facilitates targeted delivery to increase the accumulation of drugs and genes at targeted sites, thereby improving therapeutic efficacy and reducing side effects. The following section outlines several specific applications of nanotechnology in drug and gene delivery.

Nanocarriers have demonstrated tremendous potential in the delivery of small-molecule drugs and anticancer genes [115]. Conventional drug delivery systems are frequently constrained by low bioavailability, poor targeting specificity, and uneven biodistribution. Through surface modification and morphological optimization, nanomaterials can substantially improve drug-loading capacity and targeting precision, thereby enabling efficient delivery. For small-molecule drugs, nanoparticles functionalized with tumor-specific ligands can achieve precise localization, reducing damage to healthy tissues while enhancing antitumor efficacy [116]. At the same time, nanocarriers also play a pivotal role in the delivery of anticancer genes, such as p53 and p21. A major challenge in gene therapy lies in gene stability and targeting efficiency, whereas nanotechnology provides protective mechanisms that effectively prevent gene degradation in vivo [117]. Moreover, nanomaterials facilitate transmembrane transport of genetic material, thereby improving cellular uptake and further enhancing gene therapy outcomes. Recent studies have shown that LNPs can co-deliver STING agonists and mutant KRAS mRNA, effectively reprogramming the tolerogenic immune response in pancreatic cancer metastasis and significantly improving therapeutic outcomes [118]. Therefore, by optimizing the design of nanocarriers to enable precise release of drugs or genes within the tumor microenvironment, therapeutic efficacy can be markedly enhanced, particularly in the context of targeted and personalized cancer therapies.

RNA interference (RNAi) technology holds tremendous potential in cancer therapy, particularly in the regulation of tumor gene expression. Small interfering RNA (siRNA) and microRNA (miRNA) can specifically silence oncogenes, thereby effectively suppressing tumor growth, invasion, and metastasis. However, the clinical application of RNAi continues to face dual challenges of low delivery efficiency and poor molecular stability [119]. Nanocarriers provide an ideal solution for RNAi delivery. Through the rational design of nanoparticles or nanocapsules, siRNA or miRNA can be efficiently encapsulated and protected from degradation in vivo, thus ensuring their successful delivery to target cells [120,121]. In the context of TME modulation, RNAi can target specific tumor-related genes to reprogram gene expression profiles, thereby inhibiting tumor progression and metastasis. Surface-modified nanomaterials further enhance the targeting specificity of siRNA and miRNA, reduce systemic toxicity, and improve overall therapeutic efficacy. A nanoboron formulation delivering PD-L1 siRNA has been shown to combine Boron Neutron Capture Therapy (BNCT) with immunotherapy, producing synergistic antitumor effects and markedly suppressing tumor growth and metastasis in triple-negative breast cancer models [122]. By integrating RNAi with tumor-specific ligand modifications, nanocarriers enable precise delivery and efficient gene regulation, opening new avenues for individualized and comprehensive cancer therapy.

As noted in section 2.1, excessive ECM deposition and cross-linking not only drive tumor invasion and metastasis but also form major barriers to drug penetration and therapeutic efficacy. Beyond supporting tumor progression, fibrosis and ECM remodeling are closely linked to the development of therapeutic resistance [123]. Consequently, targeting these stromal components has emerged as an important therapeutic strategy. Recent advances in nanotechnology have enabled ECM-specific interventions that selectively bind to matrix molecules such as collagen and fibronectin, delivering antifibrotic agents or matrix-degrading enzymes directly to tumor sites [[124], [125], [126]]. By degrading over-deposited ECM, these nanoplatforms can disrupt stromal barriers, improve vascular perfusion, and markedly enhance intratumoral drug delivery and overall treatment outcomes [127]. A nanoplatform integrating quercetin liposomes with HAase has been shown to degrade hyaluronic acid (HA) in pancreatic ductal adenocarcinoma (PDAC) organoids, thereby enhancing matrix permeability; in vivo studies further validated its capacity to overcome stromal barriers to drug delivery and to achieve potent antitumor efficacy [128]. In addition, studies have shown that chemotherapy increases the elasticity and stiffness of the tumor ECM, which drives invasive phenotypes and induces resistance to immunotherapy [35,129]. To address this, a research team developed the nanocomposite hydrogel LOS&FeOX@Gel, which enables sustained release of losartan to reduce ECM deposition and solid stress, thereby enhancing the efficacy of oxaliplatin and markedly improving the sensitivity to checkpoint blockade therapy, ultimately achieving synergistic antimetastatic effects [129]. In summary, ECM-targeted nanointerventions represent a promising approach to overcome stromal barriers and enhance the therapeutic efficacy of cancer treatment.

Collectively, nanotechnology-based delivery strategies improve tumor targeting and efficacy while overcoming conventional therapy limitations through RNAi and ECM remodeling, thereby offering new avenues for personalized and integrated cancer treatment.

### 4.2 Nanotechnology-enabled modulation of the tumor immune microenvironment

Cancer immunotherapy represents one of the most groundbreaking advances in recent oncology treatment. The application of nanotechnology in this field offers new opportunities to enhance therapeutic efficacy and reduce adverse effects. Through nanomaterial-based drug loading and delivery systems, it is possible to precisely modulate the tumor immune microenvironment, activate immune responses, and improve overall treatment outcomes. The following summarizes several key applications of nanotechnology-mediated interventions in tumor immunotherapy.

Nanovaccines employ nanomaterial-based carriers to deliver tumor-associated antigens, thereby activating the host immune system to recognize and eliminate tumor cells [130,131]. This strategy not only significantly improves antigen stability and prolongs its half-life in vivo but also facilitates efficient presentation within the immune system [132]. Nanocarriers can enable sustained antigen release at tumor sites or within lymph nodes, thereby enhancing antigen-specific immune responses [133,134]. Through surface modifications, nanomaterials can further improve the targeting efficiency of antigen delivery, minimize nonspecific immune responses in normal tissues, and reduce associated side effects. Moreover, nanovaccines can interact with immune cells such as dendritic cells and T cells, effectively initiating and amplifying antitumor immune responses, thus providing durable and potent support for cancer immunotherapy [135]. Evidence shows that LNP composition strongly influences immune responses in mRNA vaccine delivery. A newly developed formulation, C10 LNP, induces both Th1 and Th2 responses and demonstrates potent antitumor efficacy in melanoma models [136]. Collectively, nanovaccines integrate efficient delivery, precise targeting, and immune amplification, laying a solid foundation for safer and more effective cancer immunotherapy.

Tumor cells often evade immune surveillance by activating immune checkpoint pathways, thereby weakening host immune attacks. Nanomaterials can serve as targeted delivery platforms for immune checkpoint inhibitors (such as antibodies against PD-1, PD-L1, and CTLA-4), enabling their precise localization within the tumor microenvironment and enhancing antitumor immune responses [137]. Engineered nanocarriers can efficiently bind to receptors on tumor cells, blocking immunosuppressive interactions between tumor and immune cells and restoring T-cell activity [129,138]. A recent study designed controllable antibody-producing and releasing nanoparticles (CAPRN) that accumulate in tumors via pH-responsive PEG shedding and deliver anti–PD-L1 antibody genes. Combined with photodynamic therapy, this system enhanced checkpoint inhibitor efficacy while minimizing systemic immunosuppression and toxicity [111]. This strategy highlights the significant potential of nanoplatforms in improving the safety and effectiveness of immunotherapy.

Immune cells within the TME, such as macrophages and dendritic cells, play pivotal roles in immune evasion [139]. Nanotechnology can modulate their functions through the precise delivery of immunostimulatory agents, thereby enhancing antitumor immune responses [140]. Nanomaterials are capable of inducing macrophage polarization toward a pro-inflammatory M1-like phenotype, which strengthens their phagocytic activity against tumor cells [141,142], while also augmenting the antigen-presenting capacity of dendritic cells to promote T-cell activation [143,144]. Recent studies showed that a PSA-CHD-AuNP nanoplatform efficiently delivers the R848 agonist with high tumor accumulation and prolonged retention. In combination with PD-L1 blockade and radiotherapy, it suppressed primary tumors and enhanced radioimmunotherapy against distant colorectal lesions [145]. Collectively, these advances highlight that nanoplatforms can remodel the immune cell landscape within the TME, providing strong support for improving the efficacy of tumor immunotherapy.

**Fig. 5:**
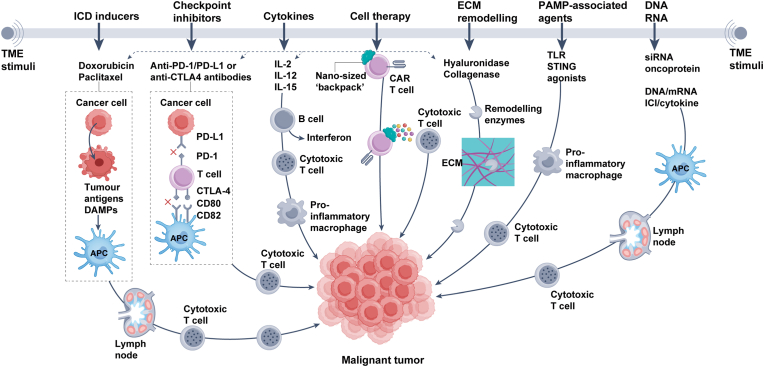
Incorporating drug cargoes into nanomaterials for cancer immunotherapy. This panel summarizes tumor-responsive nanomaterial strategies for precision immunotherapy, enabling targeted delivery of diverse agents: chemotherapeutics inducing immunogenic cell death (ICD); antigen/DAMP inducers to activate APCs; immune checkpoint inhibitors (antibodies, aptamers, small molecules, siRNAs); cytokines or cytokine plasmids; therapeutic cells with nanoscale “backpacks”; ECM-modifying agents; immunomodulators such as TLR/STING agonists; and nucleic acid therapeutics for gene silencing or protein delivery. The color scheme of this figure was partially adapted from references [14,124,228].

In conclusion, nanotechnology-based interventions—through targeted delivery, immune cell modulation, and antigen presentation—enhance immunotherapy efficacy while minimizing adverse effects, offering precise and efficient strategies with broad clinical potential (Fig. 5).

### 4.3 Nanotechnology-enabled physical therapies for TME regulation

The combination of physical therapy and TME modulation, enabled by nanotechnology, has emerged as a crucial complementary strategy in cancer treatment [146,147]. These therapies not only directly ablate tumor cells but also modulate the TME to enhance therapeutic efficacy. Representative modalities include PTT, photodynamic therapy (PDT), and magnetic hyperthermia therapy (MHT), all of which leverage nanomaterials to precisely target tumors and their microenvironment for efficient treatment.

PTT utilizes the photothermal conversion properties of nanomaterials to generate localized hyperthermia under laser irradiation at specific wavelengths, thereby achieving tumor ablation [148,149]. Nanomaterials such as gold nanoparticles and carbon nanotubes can efficiently absorb light and convert it into thermal energy, elevating the local temperature within tumors to directly kill cancer cells [150,151]. Compared with conventional hyperthermia, PTT offers superior specificity and precision; leveraging the targeting capabilities of photoresponsive nanomaterials, localized heating can be achieved at tumor sites, thereby minimizing damage to surrounding healthy tissues [152]. In addition, PTT can improve oxygen supply within the tumor region and modulate the TME, which in turn enhances the efficacy of chemotherapy and radiotherapy. Polydopamine (PDA) has been used to reduce HAuCl_4_ for synthesizing gold nanoclusters (AuNCs), which were incorporated into a metal–phenolic network (AuNC@PDA-Mn) for synergistic photothermal and chemodynamic therapy. The nanosystem accumulates in tumors via the EPR effect and releases Mn^2+^ in acidic TME. Under laser irradiation, AuNCs generate heat and accelerate Fenton reactions, producing hydroxyl radicals and thereby achieving a combined CDT/PTT effect that markedly inhibits tumor growth and metastasis [153]. Collectively, PTT not only enables direct tumor ablation but also remodels the TME, providing strong support for combinatorial therapies and demonstrating promising clinical translational potential.

PDT relies on the activation of photosensitizers under specific light irradiation to generate ROS, such as singlet oxygen, which oxidatively damage tumor cells and their microenvironment [154]. Nanomaterials serve as efficient carriers for photosensitizers, enabling precise delivery to tumor tissues and substantial ROS generation under laser exposure [155,156]. These ROS not only directly kill tumor cells but also disrupt tumor vasculature, thereby enhancing drug and immune cell penetration and significantly improving therapeutic outcomes [157]. A TME-adaptive nanoplatform (Ir1@FA@MOFs) was developed to switch from oxygen-dependent type II to hypoxia-tolerant type I PDT via Cu (II)/GSH regulation, enabling efficient ROS generation. Integrating folate targeting and TME responsiveness, it enhances delivery, depletes GSH, and disrupts redox homeostasis. The system induces immunogenic cell death, promotes CD8^+^ T-cell infiltration, and converts cold tumors into hot ones. In a 4T1 mouse model, it achieved a 50.86 % tumor inhibition rate, outperforming cisplatin without systemic toxicity [158]. Collectively, PDT not only exerts direct cytotoxic effects but also reprograms the TME through mechanism switching and immune remodeling, thereby overcoming clinical limitations and offering great potential for integrated cancer therapy.

MHT leverages the heating effect of magnetic nanoparticles in combination with external magnetic field guidance, offering a novel strategy for cancer treatment [159]. Magnetic nanoparticles, such as ferrite-based nanomaterials, can accumulate at tumor sites under the influence of an external magnetic field and generate localized heat upon exposure to an alternating magnetic field, thereby inducing thermal damage to tumor cells [160]. Beyond precise spatial control of heating regions, MHT also benefits from the intrinsic magnetic resonance imaging (MRI) capability of magnetic nanoparticles, enabling a theranostic (therapy and diagnosis) approach [160]. Its noninvasive and targeted nature allows for effective tumor treatment without harming normal tissues, while heat-mediated modulation of the TME further enhances the efficacy of other therapeutic modalities. A Korean research team recently developed 7 nm ultrasmall SPIONs that precisely localize brain tumors, enable noninvasive magnetic hyperthermia, and activate host immunity for a dual antitumor effect [161]. In summary, MHT represents not only a precise and efficient physical treatment strategy but also a robust approach to remodeling the TME, thereby supporting integrated anticancer therapies.

Together, physical therapies integrated with nanotechnology-driven TME modulation provide precise and effective cancer treatments. PTT, PDT, and MHT not only directly eradicate tumor cells but also remodel the microenvironment and enhance adjunctive therapies, offering novel strategies for cancer management (Table 2).

**Table 2:** Mechanisms and advantages of nanotechnology-mediated physical therapies combined with tumor microenvironment modulation.

Therapy	Principle	Representative Nanomaterials	Mechanism of Action	Tumor Microenvironment Modulation	Advantages	Refs
Photothermal Therapy (PTT)	Converts light energy (usually NIR laser) into heat to induce localized tumor ablation	Gold nanoparticles, carbon nanotubes, graphene oxide	Local hyperthermia destroys tumor cells directly; heat induces apoptosis and necrosis	Increases tumor oxygenation, enhances perfusion, improves drug/radiation efficacy	High spatial precision, minimal damage to normal tissue, synergistic with chemo/radiotherapy	[[243], [244], [245]]
Photodynamic Therapy (PDT)	Uses light-activated photosensitizers to generate reactive oxygen species (ROS) in tumor tissue	Porphyrin-loaded nanoparticles, upconversion NPs	Light activates photosensitizers to produce ROS (e.g., singlet oxygen), damaging tumor cells and vasculature	ROS disrupts vasculature, increases permeability, enhances immune cell infiltration	Low systemic toxicity, immune-stimulating potential, effective microenvironment remodeling	[[246], [247], [248]]
Magnetic Hyperthermia	Uses alternating magnetic fields to heat magnetic nanoparticles localized at the tumor site	Iron oxide nanoparticles (Fe_3_O_4_), ferrites	Magnetic NPs convert AC magnetic field energy into heat, causing local hyperthermia and cell death	Heat improves perfusion, increases immune response, may trigger heat shock protein expression aiding immunotherapy	Non-invasive, MRI-visible, high tumor targeting efficiency, combinable with other therapies	[159,160,249]

### 4.4 ECM-targeting nanotechnology for barrier remodeling

The ECM within the TME plays a pivotal role in tumor initiation, progression, and metastasis. Excessive deposition of ECM components such as collagen and hyaluronic acid forms a dense stromal barrier that severely impedes the effective penetration of therapeutic agents and immune cells [162]. Nanozymes, owing to their intrinsic catalytic activity, provide a novel strategy to overcome this limitation by efficiently degrading ECM components, thereby disrupting the barrier and enhancing drug permeability at tumor sites [163]. A recent study reported a ROS-responsive hyperbranched polyglycerol nanozyme (SP-NE) crosslinked with diacetylene oxalate, designed to co-deliver collagenase and a pH-sensitive paclitaxel prodrug. Upon ROS stimulation, SP-NE disassembles, releasing collagenase to weaken ECM mechanics, followed by intracellular release of paclitaxel. This system, the first to couple TME remodeling with chemotherapy, achieved synergistic antitumor effects and significantly enhanced therapeutic efficacy [164]. In summary, ECM-targeted interventions based on nanozymes offer a promising strategy to improve drug delivery efficiency and optimize combination cancer therapies.

By remodeling the ECM through nanozyme intervention, the structural features of the TME can be optimized, leading to significantly improved drug penetration. This strategy increases the local concentration of anticancer agents and thereby enhances their therapeutic efficacy [165]. In addition, ECM degradation can augment the infiltration capacity of immune cells, such as T cells and macrophages, further boosting the effectiveness of tumor immunotherapy [166].

In summary, ECM-targeted interventions using nanozymes not only overcome stromal barriers to improve drug penetration but also enhance immune cell infiltration, offering a novel strategy for integrated cancer therapy.

### 4.5 Multifunctional nanoplatforms for integrated TME modulation

Nanoplatforms for combination therapy represent an innovative strategy that integrates multiple therapeutic modalities with imaging technologies, thereby significantly enhancing cancer treatment outcomes [167,168]. Among them, theranostic nanoplatforms that combine multi-modal imaging and therapeutic functions enable real-time monitoring and precise intervention. A research team developed a pH-responsive amphiphilic photosensitizer with NIR-II fluorescence and photoacoustic imaging capabilities, demonstrating strong PDT efficacy. Coordinating with Fe^3+^ under acidic conditions restores photosensitivity, while integration of a pH-responsive polymer and GPC-3-targeting peptide enables tumor-specific activation with an on/off switch. In vitro and in vivo studies confirmed that this nanoplatform provides multimodal imaging and synergistically induces PDT and ferroptosis in hepatocellular carcinoma models [169]. In summary, such multifunctional theranostic platforms offer strong support for precision oncology and personalized treatment, while exhibiting substantial potential for clinical translation.

Multi-target combination therapy is another synergistic strategy enabled by nanoplatforms, which can simultaneously modulate multiple key aspects of the tumor microenvironment to improve therapeutic outcomes [170,171]. For example, a single nanoplatform can co-deliver chemotherapeutic agents, immunomodulators, and anti-angiogenic factors to collectively inhibit tumor growth, metastasis, and immune evasion [172,173]. This multi-targeted approach not only effectively suppresses rapid tumor progression but also enhances the durability and efficacy of treatment through comprehensive modulation of the tumor microenvironment. Moreover, the intrinsic targeting capabilities of nanocarriers ensure that these multifaceted therapies act selectively on tumor sites, thereby reducing off-target effects and improving clinical efficacy.

Overall, multifunctional nanoplatforms that integrate imaging, therapeutic, and multi-target regulatory functions provide a powerful tool for precision oncology. By enabling real-time monitoring, synergistic treatment, and comprehensive modulation of the tumor microenvironment, these platforms not only enhance therapeutic efficacy but also reduce systemic toxicity, highlighting their broad potential for clinical translation.

### 4.6 Advanced nanoplatform technologies in emerging cancer therapies

Building upon the above strategies for TME modulation, it is equally important to recognize that nanotechnology is not limited to overcoming intrinsic TME barriers. Recent advances highlight its growing integration with emerging therapeutic modalities, offering synergistic opportunities that go beyond conventional drug delivery and microenvironmental remodeling. In particular, antibody-drug conjugates (ADCs), proteolysis-targeting chimeras (PROTACs), and CRISPR-Cas9 gene-editing systems represent three frontier technologies where nanoplatforms can significantly expand therapeutic scope and improve clinical translation.

ADCs achieve selective tumor killing by conjugating monoclonal antibodies with cytotoxic agents. However, their clinical efficacy is often limited by insufficient tumor penetration, heterogeneous antigen expression, and systemic toxicity caused by premature drug release. Nanoplatforms can improve the stability and pharmacokinetic properties of ADCs, as well as enhance their intratumoral distribution. For example, liposomal and polymeric nanoparticles can serve as carriers to increase tumor accumulation while reducing off-target toxicity. In addition, they can enable the co-delivery of ADCs with synergistic agents, thereby enhancing therapeutic efficacy and overcoming drug resistance [19,[174], [175], [176], [177]].

PROTACs are bifunctional molecules that induce targeted protein degradation via the ubiquitin-proteasome system, demonstrating great promise in precisely targeting previously “undruggable” proteins [178,179]. Nevertheless, their clinical application is hindered by poor solubility, limited metabolic stability, and insufficient tumor enrichment. Nanocarrier-based delivery provides a promising solution by significantly improving the solubility and systemic stability of PROTACs, while enhancing their selective accumulation in tumor tissues [[180], [181], [182]]. In particular, stimuli-responsive nanoplatforms can achieve spatiotemporally controlled release in tumor-specific microenvironments, thereby reducing off-target effects and improving therapeutic precision.

CRISPR-Cas9 gene-editing technology offers a revolutionary approach to cancer therapy through precise genomic modification. However, its clinical translation has been hampered by challenges in safe and efficient in vivo delivery, as naked Cas9 nucleases or guide RNAs are prone to degradation and may elicit immune responses [183]. In recent years, LNPs, polymeric carriers, and metal- or silica-based nanomaterials have been widely employed to encapsulate Cas9 mRNA, plasmids, or RNP complexes, achieving efficient tumor-targeted delivery while minimizing immunogenicity [[184], [185], [186], [187]]. These systems can directly edit tumor or immune cells within the TME, for example by restoring tumor suppressor gene function, silencing oncogenes, or enhancing immune cell activity, thereby markedly improving therapeutic precision.

In summary, nanoplatforms extend beyond TME remodeling to frontier therapies such as ADCs, PROTACs, and CRISPR-Cas9, thereby overcoming traditional bottlenecks and opening new avenues in precision oncology. Although challenges remain—such as scalable manufacturing, long-term safety, and regulatory approval—advances in standardized platforms, clinical validation, and AI-driven design are expected to accelerate their translation, positioning multifunctional nanomedicine as a key driver of future precision cancer therapy.

## 5 Possibility of nanointerventions to construct an ecosystem for tumor therapy

### 5.1 Development of the concept of “ecosystem” therapy

The traditional approach to tumor treatment typically focuses on single-target therapies, such as targeting tumor cells themselves or specific molecular markers on tumor cells. However, the complexity and heterogeneity of tumors often limit the effectiveness of single-target treatments, to which cells are prone to develop resistance. Consequently, the concept of ecosystem-based therapy has introduced a new approach, which emphasizes the TME as the core and coordinates the overall regulation of immune, metabolic, and stromal components. The TME includes not only tumor cells but also immune cells, blood vessels, fibrotic tissue, and metabolic products. These factors synergistically control tumor growth, metastasis, and the response to therapy. Comprehensively regulating multiple aspects of the TME can increase the effectiveness of therapy to improve outcomes and prevent recurrence.

**Fig. 6:**
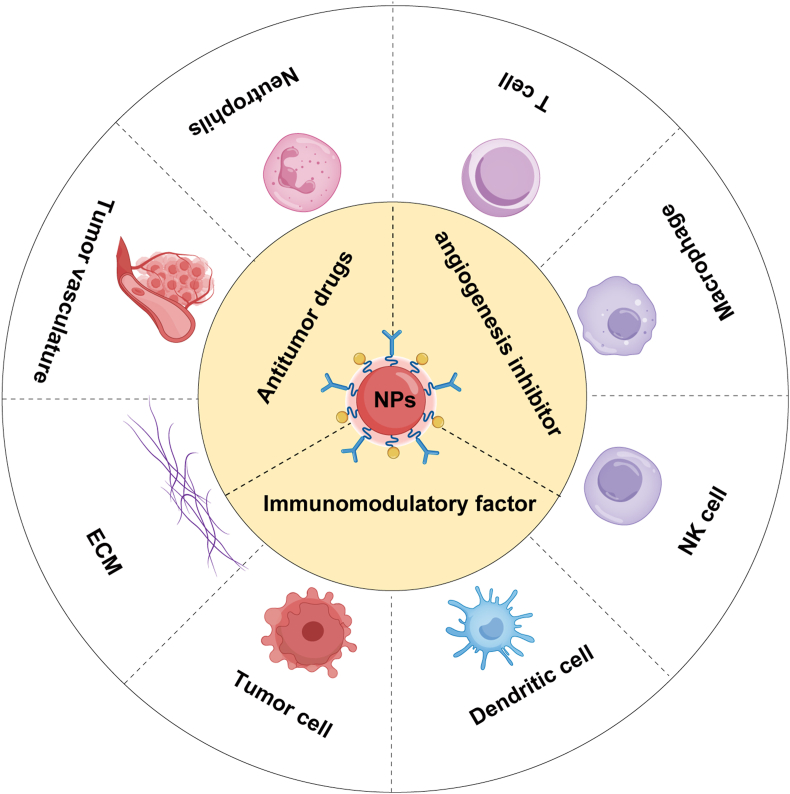
Nanointervention for constructing a therapeutic tumor ecosystem. Nanocarriers enable co-delivery of antitumor drugs, immunomodulators, and angiogenesis inhibitors, achieving synergistic effects by modulating tumor cells, reprogramming immune functions, suppressing abnormal vasculature, and remodeling the stroma. This strategy blocks immune evasion pathways (e.g., PD-1/PD-L1, Treg suppression) while reshaping the immunosuppressive TME through DC activation, CD8^+^ T cell infiltration, and M1 macrophage polarization. By integrating spatiotemporally controlled release with TME-responsive regulation, multimodal nanoplatforms offer innovative solutions for precision immunotherapy. The color scheme of this figure was partially adapted from references [[229], [230], [231], [232], [233]].

The core of this concept is shifting from single-target therapy to a systemic therapeutic approach. The use of nanotechnology as a carrier of systemic therapy facilitates simultaneous intervention at multiple targets [188]. For example, nanocarriers can simultaneously deliver anticancer drugs, immunomodulatory factors, angiogenesis inhibitors, and other therapeutic agents, coordinating tumor cells, immune cells, tumor vasculature, and stromal components to work synergistically to increase treatment efficacy [189,190]. Such multifaceted interventions not only more effectively counter various tumor escape mechanisms but also enhance immune responses and remodel the tumor microenvironment, thus providing a more comprehensive solution for cancer treatment (Fig. 6).

In summary, ecosystem-based therapy overcomes the limitations of single-target approaches by holistically regulating the tumor microenvironment. With nanotechnology as a core tool, it enables multi-level synergistic interventions and lays the groundwork for systematic and precise therapeutic ecosystems.

### 5.2 Key technologies for constructing therapeutic ecosystems

The key to constructing an ecosystem for tumor treatment lies in the integration and innovation of technologies. Multifunctional nanoplatforms serve as the core tool of this ecosystem, enabling dynamic regulation and multimode collaboration. Through the application of nanotechnology, drugs, immune factors, imaging probes, and other therapeutic molecules can be integrated into a single platform, resulting in the formation of a highly controllable system [19,191]. These nanoplatforms not only enable precise intervention at multiple targets within the tumor microenvironment but can also work synergistically under various treatment modes, such as combining phototherapy, thermotherapy, chemotherapy, and other therapeutic approaches. By acting through multiple mechanisms, they achieve more effective tumor treatment. Dynamic regulation means that treatment strategies can be adjusted in real time on the basis of changes in the tumor microenvironment, addressing tumor heterogeneity and dynamic changes.

The integration of AI technology can further increase treatment efficacy and precision. AI can assist in optimizing treatment plans, analyzing complex tumor data, and providing personalized customization. AI deep learning and big data analysis facilitate comprehensive evaluation from patient genomics and imaging data to treatment responses, offering precise and personalized decision support for cancer treatment [192]. AI can also monitor the patient’s treatment progress in real time, automatically adjusting the treatment plan to ensure the best therapeutic outcome [[193], [194], [195]]. This intelligent treatment optimization not only increases the success rate of treatment but also reduces unnecessary side effects and resource wastage. In recent years, the application of AI in nanodrug design and delivery system optimization has gradually moved from theoretical exploration to practical implementation. Studies have shown that machine learning and deep learning models can predict the relationships between nanoparticle size, surface charge, and surface modifications with their in vivo distribution, drug release kinetics, and tumor penetration capacity, thereby enabling the selection of structural parameters more suitable for penetrating the tumor stroma and cellular membranes at the design stage [68,[196], [197], [198]]. In practical applications, AI has been employed to optimize the formulation ratios of LNPs, resulting in markedly improved delivery efficiency and stability of nucleic acid therapeutics such as mRNA compared with conventional methods [[199], [200], [201], [202]]. In addition, AI-driven high-throughput screening technologies have been applied to the optimization of polymeric nanocarriers, enhancing their circulation half-life and tumor selectivity [203]. These practical cases collectively demonstrate that AI is emerging as a critical tool for advancing the precision and intelligence of nanomedicine.

In addition, data-driven precision treatment ecosystem models lay the foundation for a new paradigm in cancer therapy. By integrating clinical data, genomic information, treatment responses, and microenvironmental characteristics, individualized predictive models can be established to simulate the effects of different therapeutic regimens on tumors and their microenvironments, thereby optimizing efficacy and reducing risks [204,205]. A recent study developed an interpretable radiomics model using CT/ultrasound and machine learning to non-invasively predict nanomedicine accumulation, achieving an AUROC of 0.851. High-accumulation tumors showed 2.69-fold greater drug enrichment than low-accumulation ones, and the analysis revealed a strong link between tumor stiffness (collagen deposition) and drug delivery efficiency, providing new insights for patient stratification [206]. In summary, the integration of nanoplatforms with AI and the application of data-driven precision treatment ecosystem models will drive cancer therapy toward a more systematic, personalized, and intelligent era.

Overall, the integration of multifunctional nanoplatforms, artificial intelligence, and data-driven models is redefining cancer therapy, bridging innovation and clinical practice to enable more precise, efficient, and personalized treatments with strong translational potential.

### 5.3 Synergistic optimization with conventional treatment modalities

The combined application of nanotechnology and traditional therapies has emerged as an important strategy in cancer treatment, significantly enhancing therapeutic efficacy. Nanotechnology can be integrated with chemotherapy, radiotherapy, and targeted therapy to achieve synergistic effects [207]. For instance, nanocarriers can improve the accumulation of chemotherapeutic drugs within tumor tissues while reducing toxicity to normal tissues [[208], [209], [210]]; in radiotherapy, nanomaterials can increase the radiosensitivity of tumor cells, thereby improving therapeutic outcomes [211]; in addition, nanotechnology can be employed to deliver antibodies or small molecules precisely to key signaling pathways of tumor cells, enabling more effective intervention [116,212]. A team led by Professor Quynh-Thu Le at Stanford University recently developed a metabolizable bifunctional gold nanocluster platform (BSCgal) that integrates radiosensitization with immune checkpoint silencing. In head and neck cancer models, it markedly enhanced radio-immunotherapy efficacy, surpassing cisplatin and PD-1 inhibitors [213]. In summary, the integration of nanotechnology with conventional therapies not only improves precision and efficacy but also overcomes the limitations of single-mechanism treatments, offering new opportunities to improve cancer prognosis.

Moreover, nanointervention can reduce the side effects of traditional therapies through synergistic mechanisms. While chemotherapy and radiotherapy kill tumor cells, they are often associated with severe systemic side effects, such as immunosuppression and bone marrow suppression. Nanocarriers can precisely deliver drugs and therapeutic agents to the tumor area, avoiding damage to normal cells and thereby reducing side effects. Additionally, nanotechnology can increase the drug uptake capacity of tumor cells, improving treatment efficacy. These synergistic mechanisms ensure that the combination of traditional therapies with nanotechnology not only increases efficacy but also maximizes the protection of healthy tissues, improving the patient’s quality of life. Therefore, the integration of nanointervention with traditional treatment methods provides a more comprehensive and optimized solution for cancer therapy.

Altogether, these advances highlight that integrating nanotechnology with conventional therapies not only optimizes efficacy and safety but also paves the way for next-generation precision oncology.

## 6 Conclusion and perspective

The TME plays a pivotal role in tumor initiation, progression, and therapeutic resistance. Conventional therapies primarily target tumor cells but often neglect the complexity and dynamic nature of the TME, thereby limiting efficacy. Nanotechnology offers a promising avenue for precise TME modulation. Leveraging the EPR effect, nanocarriers enhance drug accumulation at tumor sites while reducing systemic toxicity and improving bioavailability. Beyond delivery, smart nanomaterials can remodel the TME by alleviating hypoxia, modulating acidosis, and suppressing inflammation, thereby restoring immune surveillance and overcoming limitations of traditional treatments. Furthermore, nanoplatforms enable multimodal strategies, including targeted drug delivery, immunotherapy, gene editing, and photothermal/photodynamic therapies. For instance, nanosystems have been applied to deliver immune checkpoint inhibitors and CRISPR-Cas9 tools, achieving higher precision and improved therapeutic efficacy.

Nevertheless, the clinical translation of nanomedicines remains hampered by major challenges. Their biodistribution, metabolism, and long-term safety are still incompletely understood, with most data derived from animal studies or small-scale clinical trials, lacking large-scale and long-term follow-up to assess risks such as chronic accumulation, immune activation, or genotoxicity. Hence, biosafety must be regarded as a core component of the therapeutic ecosystem. Inorganic nanocarriers provide strong stability and multifunctionality but suffer from poor biodegradability and the risk of organ accumulation, potentially causing chronic toxicity or inflammation. Organic nanocarriers display superior biocompatibility and degradability, and several have been clinically approved, yet their limited physical stability, rapid clearance by the mononuclear phagocyte system, and batch-to-batch variability remain obstacles for industrialization. Hybrid nanocarriers combine the advantages of inorganic and organic systems and are particularly promising for multifunctional and stimuli-responsive applications; however, their complex synthesis, high production cost, and lack of long-term biosafety data pose significant hurdles. Therefore, comprehensive toxicological evaluation, long-term clinical monitoring, and the establishment of standardized quality control and internationally harmonized safety frameworks are indispensable to advance industrialization and regulatory approval.

Against this backdrop, AI offers transformative opportunities to address existing bottlenecks. Machine learning can predict how nanoparticle size, charge, and surface modifications affect circulation and tumor penetration, accelerating preclinical design and optimization. Deep learning has already been used to refine lipid nanoparticle formulations, markedly enhancing the stability and efficiency of nucleic acid delivery. AI-driven modeling further integrates multi-omics data with TME-specific parameters to guide the rational design of stimuli-responsive nanoplatforms. In addition, digital twins and adaptive AI systems enable real-time simulation of patient responses and dynamic adjustment of dosing regimens, thereby improving efficacy while reducing toxicity. Coupled with automated manufacturing and quality control, AI also minimizes batch variability, enhances scalability, and streamlines regulatory evaluation, collectively accelerating clinical translation.

Looking forward, the evolution of intelligent, adaptive, and multifunctional nanoplatforms will be pivotal for precision oncology. Integrated theranostic systems not only enable real-time monitoring of drug distribution and efficacy but also improve treatment safety and accuracy. Building such a systematic, patient-centered therapeutic ecosystem requires close collaboration across materials science, oncology, computational biology, bioinformatics, and regulatory science. Despite challenges in biosafety validation, scalable production, quality control, and policy harmonization, the convergence of nanotechnology with AI and big data—supported by standardized safety evaluation and regulatory frameworks—holds great promise to accelerate the clinical translation of nanomedicine, ultimately delivering safer, more effective, and personalized therapies to improve patient outcomes and quality of life.

## CRediT authorship contribution statement

**Xiangying Deng:** Writing – review & editing, Writing – original draft, Software, Funding acquisition, Formal analysis. **Xinglong Liu:** Software, Resources, Formal analysis. **Lin Zhao:** Writing – review & editing, Writing – original draft, Supervision, Software, Funding acquisition.

## Ethical statement

Not applicable.

## Consent for publication

All authors consent for publication of this review.

## Funding

This study was funded by the 10.13039/501100001809National Natural Science Foundation of China (82103653 and 82303258), the 10.13039/501100004735Natural Science Foundation of Hunan Province (No. 2022JJ40659 and No. 2023JJ40874), the Scientific Research Launch Project for new employees of the Second Xiangya Hospital of Central South University (QH20230202).

## Declaration of competing interest

The authors declare the following financial interests/personal relationships which may be considered as potential competing interests: Lin Zhao reports financial support was provided by National Natural Science Foundation of China. Xingying Deng reports financial support was provided by National Natural Science Foundation of China. Lin Zhao reports financial support was provided by Natural Science Foundation of Hunan Province. Xiangying Deng reports financial support was provided by Natural Science Foundation of Hunan Province. If there are other authors, they declare that they have no known competing financial interests or personal relationships that could have appeared to influence the work reported in this paper.

## Data Availability

No data was used for the research described in the article.

## References

[1] Soerjomataram I., Bray F. (2021). Planning for tomorrow: global cancer incidence and the role of prevention 2020-2070. Nat. Rev. Clin. Oncol..

[2] Wang D., Liu B., Zhang Z. (2023). Accelerating the understanding of cancer biology through the lens of genomics. Cell.

[3] Perez-Gonzalez A., Bevant K., Blanpain C. (2023). Cancer cell plasticity during tumor progression, metastasis and response to therapy. Nat. Cancer.

[4] Janjigian Y.Y., Wolchok J.D., Ariyan C.E. (2021). Eradicating micrometastases with immune checkpoint blockade: strike while the iron is hot. Cancer Cell.

[5] Liu Y.Q., Wang X.L., He D.H., Cheng Y.X. (2021). Protection against chemotherapy- and radiotherapy-induced side effects: a review based on the mechanisms and therapeutic opportunities of phytochemicals. Phytomedicine.

[6] Weiss F., Lauffenburger D., Friedl P. (2022). Towards targeting of shared mechanisms of cancer metastasis and therapy resistance. Nat. Rev. Cancer.

[7] Stoletov K., Beatty P.H., Lewis J.D. (2020). Novel therapeutic targets for cancer metastasis. Expert Rev. Anticancer Ther..

[8] Ren X., Zhang L., Zhang Y., Li Z., Siemers N., Zhang Z. (2021). Insights gained from single-cell analysis of immune cells in the tumor microenvironment. Annu. Rev. Immunol..

[9] de Visser K.E., Joyce J.A. (2023). The evolving tumor microenvironment: from cancer initiation to metastatic outgrowth. Cancer Cell.

[10] Sattiraju A., Kang S., Giotti B., Chen Z., Marallano V.J., Brusco C. (2023). Hypoxic niches attract and sequester tumor-associated macrophages and cytotoxic T cells and reprogram them for immunosuppression. Immunity.

[11] Bai R., Li Y., Jian L., Yang Y., Zhao L., Wei M. (2022). The hypoxia-driven crosstalk between tumor and tumor-associated macrophages: mechanisms and clinical treatment strategies. Mol. Cancer.

[12] Jin R., Neufeld L., McGaha T.L. (2025). Linking macrophage metabolism to function in the tumor microenvironment. Nat. Cancer.

[13] Chen D., Zhang X., Li Z., Zhu B. (2021). Metabolic regulatory crosstalk between tumor microenvironment and tumor-associated macrophages. Theranostics.

[14] Zhang P., Meng J., Li Y., Yang C., Hou Y., Tang W. (2021). Nanotechnology-enhanced immunotherapy for metastatic cancer. Innovation.

[15] Jia X., Wang Y., Qiao Y., Jiang X., Li J. (2024). Nanomaterial-based regulation of redox metabolism for enhancing cancer therapy. Chem. Soc. Rev..

[16] Mendes B.B., Conniot J., Avital A., Yao D., Jiang X., Zhou X. (2022). Nanodelivery of nucleic acids. Nat. Rev. Method. Prim..

[17] Yang X., Huang C., Wang H., Yang K., Huang M., Zhang W. (2024). Multifunctional nanoparticle-loaded injectable alginate hydrogels with deep tumor penetration for enhanced chemo-immunotherapy of cancer. ACS Nano.

[18] Zhu X., Li S. (2023). Nanomaterials in tumor immunotherapy: new strategies and challenges. Mol. Cancer.

[19] Wang B., Hu S., Teng Y., Chen J., Wang H., Xu Y. (2024). Current advance of nanotechnology in diagnosis and treatment for malignant tumors. Signal Transduct. Targeted Ther..

[20] Ma S., Xu W., Fei Y., Li D., Jia X., Wang J. (2023). Mn(2+)/Ir(3+) -Doped and CaCO(3) -Covered prussian blue nanoparticles with indocyanine green encapsulation for tumor microenvironment modulation and image-guided synergistic cancer therapy. Adv. Healthcare Mater..

[21] Linderman S.W., DeRidder L., Sanjurjo L., Foote M.B., Alonso M.J., Kirtane A.R. (2025). Enhancing immunotherapy with tumour-responsive nanomaterials. Nat. Rev. Clin. Oncol..

[22] Lu Q., Kou D., Lou S., Ashrafizadeh M., Aref A.R., Canadas I. (2024). Nanoparticles in tumor microenvironment remodeling and cancer immunotherapy. J. Hematol. Oncol..

[23] Liu Z., Wang Z., Zhang Z., Zhang Z., Qi X., Zhu H. (2024). Engineering nanosensitizer to remodel the TME for hypoimmunogenic “Cold”-“Hot” tumor transformations. Nano Lett..

[24] Toledo B., Zhu Chen L., Paniagua-Sancho M., Marchal J.A., Peran M., Giovannetti E. (2024). Deciphering the performance of macrophages in tumour microenvironment: a call for precision immunotherapy. J. Hematol. Oncol..

[25] Ding C., Shrestha R., Zhu X., Geller A.E., Wu S., Woeste M.R. (2023). Inducing trained immunity in pro-metastatic macrophages to control tumor metastasis. Nat. Immunol..

[26] Bejarano L., Jordao M.J.C., Joyce J.A. (2021). Therapeutic targeting of the tumor microenvironment. Cancer Discov..

[27] Guan F., Wang R., Yi Z., Luo P., Liu W., Xie Y. (2025). Tissue macrophages: origin, heterogenity, biological functions, diseases and therapeutic targets. Signal Transduct. Targeted Ther..

[28] Laskowski T.J., Biederstadt A., Rezvani K. (2022). Natural killer cells in antitumour adoptive cell immunotherapy. Nat. Rev. Cancer.

[29] Xia L., Oyang L., Lin J., Tan S., Han Y., Wu N. (2021). The cancer metabolic reprogramming and immune response. Mol. Cancer.

[30] Song J., Wei R., Liu C., Zhao Z., Liu X., Wang Y. (2025). Antigen-presenting cancer associated fibroblasts enhance antitumor immunity and predict immunotherapy response. Nat. Commun..

[31] Hu D., Li Z., Zheng B., Lin X., Pan Y., Gong P. (2022). Cancer-associated fibroblasts in breast cancer: challenges and opportunities. Cancer Commun..

[32] Wang-Bishop L., Kimmel B.R., Ngwa V.M., Madden M.Z., Baljon J.J., Florian D.C. (2023). STING-activating nanoparticles normalize the vascular-immune interface to potentiate cancer immunotherapy. Sci. Immunol..

[33] Glaviano A., Lau H.S., Carter L.M., Lee E.H.C., Lam H.Y., Okina E. (2025). Harnessing the tumor microenvironment: targeted cancer therapies through modulation of epithelial-mesenchymal transition. J. Hematol. Oncol..

[34] Cambria E., Coughlin M.F., Floryan M.A., Offeddu G.S., Shelton S.E., Kamm R.D. (2024). Linking cell mechanical memory and cancer metastasis. Nat. Rev. Cancer.

[35] Sleeboom J.J.F., van Tienderen G.S., Schenke-Layland K., van der Laan L.J.W., Khalil A.A., Verstegen M.M.A. (2024). The extracellular matrix as hallmark of cancer and metastasis: from biomechanics to therapeutic targets. Sci. Transl. Med..

[36] Hu Q., Zhu Y., Mei J., Liu Y., Zhou G. (2025). Extracellular matrix dynamics in tumor immunoregulation: from tumor microenvironment to immunotherapy. J. Hematol. Oncol..

[37] Zhao H., Gao Y., Ma S., Si X., Li J., Qi Y. (2025). Hyaluronidase nanogel-armed CAR-T cell for synergistically reducing tumor extracellular matrix and improving efficacy against solid tumors. Nano Res..

[38] Chen Z., Han F., Du Y., Shi H., Zhou W. (2023). Hypoxic microenvironment in cancer: molecular mechanisms and therapeutic interventions. Signal Transduct. Targeted Ther..

[39] Zhuang Y., Liu K., He Q., Gu X., Jiang C., Wu J. (2023). Hypoxia signaling in cancer: implications for therapeutic interventions. MedComm.

[40] Chen J., Huang Z., Chen Y., Tian H., Chai P., Shen Y. (2025). Lactate and lactylation in cancer. Signal Transduct. Targeted Ther..

[41] Xu N., Zhu Y., Han Y., Liu Q., Tong L., Li Y. (2025). Targeting MondoA-TXNIP restores antitumour immunity in lactic-acid-induced immunosuppressive microenvironment. Nat. Metab..

[42] Llibre A., Kucuk S., Gope A., Certo M., Mauro C. (2025). Lactate: a key regulator of the immune response. Immunity.

[43] Zhang J., Yang C., Tan J., Liu B., Yang Z., Li Z. (2025). Bimetallic nanoadjuvant-mediated glutamine metabolism intervention and STING activation for enhanced antitumor immunity. Nano Today.

[44] Roerden M., Spranger S. (2025). Cancer immune evasion, immunoediting and intratumour heterogeneity. Nat. Rev. Immunol..

[45] Nishikawa H., Koyama S. (2021). Mechanisms of regulatory T cell infiltration in tumors: implications for innovative immune precision therapies. J. Immunother. Cancer.

[46] Li C., Jiang P., Wei S., Xu X., Wang J. (2020). Regulatory T cells in tumor microenvironment: new mechanisms, potential therapeutic strategies and future prospects. Mol. Cancer.

[47] Lasser S.A., Ozbay Kurt F.G., Arkhypov I., Utikal J., Umansky V. (2024). Myeloid-derived suppressor cells in cancer and cancer therapy. Nat. Rev. Clin. Oncol..

[48] Mempel T.R., Lill J.K., Altenburger L.M. (2024). How chemokines organize the tumour microenvironment. Nat. Rev. Cancer.

[49] Ozga A.J., Chow M.T., Luster A.D. (2021). Chemokines and the immune response to cancer. Immunity.

[50] Tiwari A., Trivedi R., Lin S.Y. (2022). Tumor microenvironment: barrier or opportunity towards effective cancer therapy. J. Biomed. Sci..

[51] Han Y., Liu D., Li L. (2020). PD-1/PD-L1 pathway: current researches in cancer. Am. J. Cancer Res..

[52] Wang F., Jin Y., Wang M., Luo H.Y., Fang W.J., Wang Y.N. (2024). Combined anti-PD-1, HDAC inhibitor and anti-VEGF for MSS/pMMR colorectal cancer: a randomized phase 2 trial. Nat. Med..

[53] Lee C., Kim M.J., Kumar A., Lee H.W., Yang Y., Kim Y. (2025). Vascular endothelial growth factor signaling in health and disease: from molecular mechanisms to therapeutic perspectives. Signal Transduct. Targeted Ther..

[54] Guelfi S., Hodivala-Dilke K., Bergers G. (2024). Targeting the tumour vasculature: from vessel destruction to promotion. Nat. Rev. Cancer.

[55] Starzer A.M., Preusser M., Berghoff A.S. (2022). Immune escape mechanisms and therapeutic approaches in cancer: the cancer-immunity cycle. Ther. Adv. Med. Oncol..

[56] Jeong H., Koh J., Kim S., Yim J., Song S.G., Kim H. (2025). Cell-intrinsic PD-L1 signaling drives immunosuppression by myeloid-derived suppressor cells through IL-6/Jak/Stat3 in PD-L1-high lung cancer. J. Immunother. Cancer.

[57] Kay E.J., Zanivan S. (2025). The tumor microenvironment is an ecosystem sustained by metabolic interactions. Cell Rep..

[58] Bakrim S., Fessikh M.E., Elhrech H., Omari N.E., Amanullah M., Ming L.C. (2025). Targeting inflammation in cancer therapy: from mechanistic insights to emerging therapeutic approaches. J. Transl. Med..

[59] Wang H., Wang T., Yan S., Tang J., Zhang Y., Wang L. (2024). Crosstalk of pyroptosis and cytokine in the tumor microenvironment: from mechanisms to clinical implication. Mol. Cancer.

[60] Nowak-Sliwinska P., van Beijnum J.R., Griffioen C.J., Huinen Z.R., Sopesens N.G., Schulz R. (2023). Proinflammatory activity of VEGF-targeted treatment through reversal of tumor endothelial cell anergy. Angiogenesis.

[61] Yan Y., Huang L., Liu Y., Yi M., Chu Q., Jiao D. (2022). Metabolic profiles of regulatory T cells and their adaptations to the tumor microenvironment: implications for antitumor immunity. J. Hematol. Oncol..

[62] Zhang T., Lin Y., Gao Q. (2023). Bispecific antibodies targeting immunomodulatory checkpoints for cancer therapy. Cancer Biol. Med..

[63] Li T., Niu M., Zhou J., Wu K., Yi M. (2024). The enhanced antitumor activity of bispecific antibody targeting PD-1/PD-L1 signaling. Cell Commun. Signal..

[64] De Martino M., Rathmell J.C., Galluzzi L., Vanpouille-Box C. (2024). Cancer cell metabolism and antitumour immunity. Nat. Rev. Immunol..

[65] Zhong Y., Geng F., Mazik L., Yin X., Becker A.P., Mohammed S. (2024). Combinatorial targeting of glutamine metabolism and lysosomal-based lipid metabolism effectively suppresses glioblastoma. Cell Rep. Med..

[66] Luo Y., Li Y., Huang Z., Li X., Wang Y., Hou J. (2022). A nanounit strategy disrupts energy metabolism and alleviates immunosuppression for cancer therapy. Nano Lett..

[67] Khizar S., Alrushaid N., Alam Khan F., Zine N., Jaffrezic-Renault N., Errachid A. (2023). Nanocarriers based novel and effective drug delivery system. Int. J. Pharm..

[68] Mendes B.B., Zhang Z., Conniot J., Sousa D.P., Ravasco J., Onweller L.A. (2024). A large-scale machine learning analysis of inorganic nanoparticles in preclinical cancer research. Nat. Nanotechnol..

[69] Qi M.H., Wang D.D., Qian W., Zhang Z.L., Ao Y.W., Li J.M. (2025). High-efficiency gold nanoaggregates for NIR LED-driven sustained mild photothermal therapy achieving complete tumor eradication and immune enhancement. Adv. Mater..

[70] Zhou D., Shan S., Chen L., Li C., Wang H., Lu K. (2024). Trapped in endosome PEGylated ultra-small iron oxide nanoparticles enable extraordinarily high MR imaging contrast for hepatocellular carcinomas. Adv. Sci. (Weinh.).

[71] Janjua T.I., Cao Y., Kleitz F., Linden M., Yu C., Popat A. (2023). Silica nanoparticles: a review of their safety and current strategies to overcome biological barriers. Adv. Drug Deliv. Rev..

[72] Tenchov R., Bird R., Curtze A.E., Zhou Q. (2021). Lipid nanoparticles horizontal line from liposomes to mRNA vaccine delivery, a landscape of research diversity and advancement. ACS Nano.

[73] Jia W., Wu Y., Xie Y., Yu M., Chen Y. (2025). Advanced polymeric nanoparticles for cancer immunotherapy: materials engineering, immunotherapeutic mechanism and clinical translation. Adv. Mater..

[74] Zang W., Gao D., Yu M., Long M., Zhang Z., Ji T. (2023). Oral delivery of gemcitabine-loaded glycocholic acid-modified micelles for cancer therapy. ACS Nano.

[75] Cui F., Garcia-Lopez V., Wang Z., Luo Z., He D., Feng X. (2025). Two-Dimensional Organic-Inorganic van der Waals Hybrids. Chem. Rev..

[76] Wang Y., Li X., Wang Y., Chen H., Gao Y., Lin Y. (2024). Facile construction of gold- and BODIPY-Coordinated liposomal nanocomplexes for enhanced photothermal therapy of cancer. Mater. Des..

[77] Xu L., Jiang X., Liang K., Gao M., Kong B. (2021). Frontier luminous strategy of functional silica nanohybrids in sensing and bioimaging: from ACQ to AIE. Aggregate.

[78] Wang H., Gao L., Fan T., Zhang C., Zhang B., Al-Hartomy O.A. (2021). Strategic design of intelligent-responsive nanogel carriers for cancer therapy. ACS Appl. Mater. Interfaces.

[79] Zhang D., Chen Y., Hao M., Xia Y. (2024). Putting hybrid nanomaterials to work for biomedical applications. Angew Chem. Int. Ed. Engl..

[80] Zhu L., Wu J., Gao H., Wang T., Xiao G., Hu C. (2023). Tumor immune microenvironment-modulated nanostrategy for the treatment of lung cancer metastasis. Chin. Med. J. (Engl)..

[81] Rad M.E., Soylukan C., Kulabhusan P.K., Gunaydin B.N., Yuce M. (2023). Material and design toolkit for drug delivery: state of the art, trends, and challenges. ACS Appl. Mater. Interfaces.

[82] Belyaev I.B., Griaznova O.Y., Yaremenko A.V., Deyev S.M., Zelepukin I.V. (2025). Beyond the EPR effect: intravital microscopy analysis of nanoparticle drug delivery to tumors. Adv. Drug Deliv. Rev..

[83] Wu J.L.Y., Ji Q., Blackadar C., Nguyen L.N.M., Lin Z.P., Sepahi Z. (2025). The pathways for nanoparticle transport across tumour endothelium. Nat. Nanotechnol..

[84] Zha S., Liu H., Li H., Li H., Wong K.L., All A.H. (2024). Functionalized nanomaterials capable of crossing the blood-brain barrier. ACS Nano.

[85] Wang Y., Baars I., Berzina I., Rocamonde-Lago I., Shen B., Yang Y. (2024). A DNA robotic switch with regulated autonomous display of cytotoxic ligand nanopatterns. Nat. Nanotechnol..

[86] Luo H., Lv J., Wen P., Zhang S., Ma W., Yang Z. (2025). Supramolecular polyrotaxane-based nano-theranostics enable cancer-cell stiffening for enhanced T-cell-mediated anticancer immunotherapy. Nat. Commun..

[87] Yi Q., Liu L., Xie G. (2024). Recent advances of stimuli-responsive liquid-liquid interfaces stabilized by nanoparticles. ACS Nano.

[88] Dosta P., Dion M.Z., Prado M., Hurtado P., Riojas-Javelly C.J., Cryer A.M. (2024). Matrix Metalloproteinase- and pH-Sensitive nanoparticle system enhances drug retention and penetration in glioblastoma. ACS Nano.

[89] Hu Z., Tan H., Ye Y., Xu W., Gao J., Liu L. (2024). NIR-actuated ferroptosis nanomotor for enhanced tumor penetration and therapy. Adv. Mater..

[90] He G., He M., Wang R., Li X., Hu H., Wang D. (2023). A near-infrared light-activated photocage based on a ruthenium complex for cancer phototherapy. Angew Chem. Int. Ed. Engl..

[91] Zhu J., Wang J., Li Y. (2023). Recent advances in magnetic nanocarriers for tumor treatment. Biomed. Pharmacother..

[92] Zhang H., Wu M., Sumadi F.A.N., Fu C., Meng Q., Alanazi M. (2024). Responsive theranostic nanoprobe for ratiometric photoacoustic monitoring of hypochlorous acid‐mediated inflammation in cancer photothermal therapy. Adv. Funct. Mater..

[93] Xu C., Qin X., Wei X., Yu J., Zhang Y., Zhang Y. (2025). A cascade X-ray energy converting approach toward radio-afterglow cancer theranostics. Nat. Nanotechnol..

[94] Gawne P.J., Ferreira M., Papaluca M., Grimm J., Decuzzi P. (2023). New opportunities and old challenges in the clinical translation of nanotheranostics. Nat. Rev. Mater..

[95] Song J., Wang H., Meng X., Li W., Qi J. (2024). A hypoxia-activated and microenvironment-remodeling nanoplatform for multifunctional imaging and potentiated immunotherapy of cancer. Nat. Commun..

[96] Rebaudi F., De Franco F., Goda R., Obino V., Vita G., Baronti C. (2024). The landscape of combining immune checkpoint inhibitors with novel therapies: secret alliances against breast cancer. Cancer Treat Rev..

[97] Liu Y., Xie Y., Chen Y., Duan J., Bao C., Wang J. (2025). A protease-cleavable liposome for co-delivery of anti-PD-L1 and doxorubicin for colon cancer therapy in mice. Nat. Commun..

[98] Chen Y., Ma W., Yu Y., Niu Q., Li Y., Yin S. (2025). AIE-augmented NIR-II-Emissive supramolecular metallacycle nanoplatform for tumor microenvironment-responsive chemo-photothermal-immunotherapy. Adv. Healthcare Mater..

[99] Chen J., Bu C., Lu Y., Peng X., Yu J., Ding X. (2025). Bioresponsive nanoreactor initiates cascade reactions for tumor vascular normalization and lactate depletion to augment immunotherapy. Biomaterials.

[100] Zhao R., Hou Y., Li B., Pan Z., Qiu J., Wang Q. (2025). Bioengineered hybrid dual-targeting nanoparticles reprogram the tumour microenvironment for deep glioblastoma photodynamic therapy. Nat. Commun..

[101] Zhou M., Feng J., Mei Q., Li T., Zhang Y., Liu W. (2025). A powerful tumor catalytic therapy by an enzyme-nanozyme Cascade catalysis (ENCAT) system. Small.

[102] Dong S., Dong Y., Liu B., Liu J., Liu S., Zhao Z. (2022). Guiding transition metal-doped hollow cerium tandem nanozymes with elaborately regulated multi-enzymatic activities for intensive chemodynamic therapy. Adv. Mater..

[103] Guo D., Tong Y., Jiang X., Meng Y., Jiang H., Du L. (2022). Aerobic glycolysis promotes tumor immune evasion by hexokinase2-mediated phosphorylation of IkappaBalpha. Cell Metab..

[104] Yang J., Su T., Wang Q., Shi R., Ding J., Chen X. (2025). Glucose metabolism-targeted Poly(amino acid) nanoformulation of Oxaliplatin(IV)-Aspirin prodrug for enhanced chemo-immunotherapy. Adv. Mater..

[105] Ma J., Guo D., Ji X., Zhou Y., Liu C., Li Q. (2023). Composite hydrogel for spatiotemporal lipid intervention of tumor milieu. Adv. Mater..

[106] Cheng X., Xu J., Cui Y., Liu J., Chen Y., He C. (2025). Nanovesicles for lipid metabolism reprogram-enhanced ferroptosis and magnetotherapy of refractory tumors and inhibiting metastasis with activated innate immunity. ACS Nano.

[107] Wu S., Xu L., He C., Wang P., Qin J., Guo F. (2023). Lactate efflux inhibition by Syrosingopine/LOD Co-Loaded nanozyme for synergetic self-replenishing catalytic cancer therapy and immune microenvironment remodeling. Adv. Sci. (Weinh.).

[108] Ma M., Li X., Zhong M., Li X., Yu J., Wang Z. (2025). Galloylated toll-like receptor 7/8 agonist nanovaccine for enhanced tumor antigen delivery in personalized immunotherapy. ACS Nano.

[109] Zhu Q., Yu C., Chen Y., Luo W., Li M., Zou J. (2025). Dual mRNA nanoparticles strategy for enhanced pancreatic cancer treatment and beta-elemene combination therapy. Proc. Natl. Acad. Sci. U. S. A..

[110] Miao Y., Niu L., Lv X., Zhang Q., Xiao Z., Ji Z. (2024). A minimalist pathogen-like sugar nanovaccine for enhanced cancer immunotherapy. Adv. Mater..

[111] Park M., Lim J., Lee S., Nah Y., Kang Y., Kim W.J. (2025). Nanoparticle-mediated explosive Anti-PD-L1 factory built in tumor for advanced immunotherapy. Adv. Mater..

[112] Regeni I., Bonnet S. (2025). Supramolecular approaches for the treatment of hypoxic regions in tumours. Nat. Rev. Chem.

[113] Deng Y., Jiang Z., Jin Y., Qiao J., Yang S., Xiong H. (2021). Reinforcing vascular normalization therapy with a bi-directional nano-system to achieve therapeutic-friendly tumor microenvironment. J. Contr. Release.

[114] Huang N., Liu Y., Fang Y., Zheng S., Wu J., Wang M. (2020). Gold nanoparticles induce tumor vessel normalization and impair metastasis by inhibiting endothelial Smad2/3 signaling. ACS Nano.

[115] Boehnke N., Straehla J.P., Safford H.C., Kocak M., Rees M.G., Ronan M. (2022). Massively parallel pooled screening reveals genomic determinants of nanoparticle delivery. Science.

[116] Mitchell M.J., Billingsley M.M., Haley R.M., Wechsler M.E., Peppas N.A., Langer R. (2021). Engineering precision nanoparticles for drug delivery. Nat. Rev. Drug Discov..

[117] Wei Hu S., Ding T., Tang H., Guo H., Cui W., Shu Y. (2023). Nanobiomaterial vectors for improving gene editing and gene therapy. Mater. Today.

[118] Xu X., Wang X., Liao Y.P., Luo L., Nel A.E. (2025). Reprogramming the tolerogenic immune response against pancreatic cancer metastases by lipid nanoparticles delivering a STING agonist plus mutant KRAS mRNA. ACS Nano.

[119] Jadhav V., Vaishnaw A., Fitzgerald K., Maier M.A. (2024). RNA interference in the era of nucleic acid therapeutics. Nat. Biotechnol..

[120] Jin Y., Zhang B., Li J., Guo Z., Zhang C., Chen X. (2025). Bioengineered protein nanocarrier facilitating siRNA escape from lysosomes for targeted RNAi therapy in glioblastoma. Sci. Adv..

[121] Zhang J., Chen B., Gan C., Sun H., Zhang J., Feng L. (2023). A comprehensive review of small interfering RNAs (siRNAs): mechanism, therapeutic targets, and delivery strategies for cancer therapy. Int. J. Nanomed..

[122] Deng S., Hu L., Chen G., Ye J., Xiao Z., Guan T. (2025). A PD-L1 siRNA-Loaded boron nanoparticle for targeted cancer radiotherapy and immunotherapy. Adv. Mater..

[123] Prakash J., Shaked Y. (2024). The interplay between extracellular matrix remodeling and cancer therapeutics. Cancer Discov..

[124] Zhang J., Hu Y., Wen X., Yang Z., Wang Z., Feng Z. (2025). Tandem-controlled lysosomal assembly of nanofibres induces pyroptosis for cancer immunotherapy. Nat. Nanotechnol..

[125] Cahn D., Stern A., Buckenmeyer M., Wolf M., Duncan G.A. (2024). Extracellular matrix limits nanoparticle diffusion and cellular uptake in a tissue-specific manner. ACS Nano.

[126] Li S., Zhang Y., Ho S.H., Li B., Wang M., Deng X. (2020). Combination of tumour-infarction therapy and chemotherapy via the co-delivery of doxorubicin and thrombin encapsulated in tumour-targeted nanoparticles. Nat. Biomed. Eng..

[127] Yao W.Q., Song W.F., Deng X.C., Lin Y.T., Meng R., Wang J.W. (2025). Harnessing the engineered probiotic-nanosystem to remodulate tumor extracellular matrix and regulate tumor-colonizing bacteria for improving pancreatic cancer chemo-immunotherapy. Small.

[128] Sun G., Wu Y., Li J., Yang M., Xu H., Li Y. (2025). Quercetin liposomes conjugated with hyaluronidase: an efficient drug delivery system to block pancreatic cancer. J. Contr. Release.

[129] Hou X., Shen Y., Huang B., Li Q., Li S., Jiang T. (2024). Losartan-based nanocomposite hydrogel overcomes chemo-immunotherapy resistance by remodeling tumor mechanical microenvironment. J. Nanobiotechnol..

[130] Zhang Y., Chen J., Shi L., Ma F. (2023). Polymeric nanoparticle-based nanovaccines for cancer immunotherapy. Mater. Horiz..

[131] Liu J., Cui Y., Cabral H., Tong A., Yue Q., Zhao L. (2024). Glucosylated nanovaccines for dendritic cell-targeted antigen delivery and amplified cancer immunotherapy. ACS Nano.

[132] Ling X., Dong Z., He J., Chen D., He D., Guo R. (2025). Advances in polymer-based self-adjuvanted nanovaccines. Small.

[133] Chen C., Xu Y., Meng H., Bao H., Hu Y., Li C. (2025). Nano-oncologic vaccine for boosting cancer immunotherapy: the Horizons in cancer treatment. Nanomaterials.

[134] Lu Y., Ma N., Cheng K., Liu G., Liang J., Xu C. (2025). An OMV-based nanovaccine as antigen presentation signal enhancer for cancer immunotherapy. Adv. Mater..

[135] Go S., Jung M., Lee S., Moon S., Hong J., Kim C. (2023). A personalized cancer nanovaccine that enhances T-Cell responses and efficacy through dual interactions with dendritic cells and T cells. Adv. Mater..

[136] Zhu Y., Ma J., Shen R., Lin J., Li S., Lu X. (2024). Screening for lipid nanoparticles that modulate the immune activity of helper T cells towards enhanced antitumour activity. Nat. Biomed. Eng..

[137] Reda M., Ngamcherdtrakul W., Nelson M.A., Siriwon N., Wang R., Zaidan H.Y. (2022). Development of a nanoparticle-based immunotherapy targeting PD-L1 and PLK1 for lung cancer treatment. Nat. Commun..

[138] Yan T., Liao Q., Chen Z., Xu Y., Zhu W., Hu P. (2025). beta-Ketoenamine covalent organic framework nanoplatform combined with immune checkpoint blockade via photodynamic immunotherapy inhibit glioblastoma progression. Bioact. Mater..

[139] Haynes N.M., Chadwick T.B., Parker B.S. (2024). The complexity of immune evasion mechanisms throughout the metastatic cascade. Nat. Immunol..

[140] Wen N., Lu Y., Zhuo Y., Fu B., Wang H., He Y. (2025). Enhancing T-Cell infiltration and immunity in solid tumors via DNA nanolinker-mediated monocyte hitchhiking. J. Am. Chem. Soc..

[141] Liu L., Fu S., Gu H., Li Y., Zhu G., Ai H. (2025). Platinum(IV)-Backboned polymer prodrug-functionalized manganese oxide nanoparticles for enhanced lung cancer chemoimmunotherapy via amplifying stimulator of interferon genes activation. ACS Nano.

[142] Gong Y., Gao W., Zhang J., Dong X., Zhu D., Ma G. (2024). Engineering nanoparticles-enabled tumor-associated macrophages repolarization and phagocytosis restoration for enhanced cancer immunotherapy. J. Nanobiotechnol..

[143] Ding J., Zhao X., Long S., Sun W., Du J., Fan J. (2025). A dual stimuli-responsive nanoimmunomodulator for antitumor synergy of macrophages and T cells. ACS Nano.

[144] Li Y., Dong Y., Shen D., Guo Y., Cao Y., Zhang K. (2025). Personalized nanovaccine based on STING-activating nanocarrier for robust cancer immunotherapy. ACS Nano.

[145] Lv Z., Li M., Zhu J., Guo Y., Zhang Y., Zhao Z. (2025). Engineering TLR7/8 agonist-loaded and tumor-anchored gold nanosensitizers for enhanced radioimmunotherapy. Small.

[146] Wang X., Zhang H., Chen X., Wu C., Ding K., Sun G. (2023). Overcoming tumor microenvironment obstacles: current approaches for boosting nanodrug delivery. Acta Biomater..

[147] Chen W., Zhang Z., Han Y., Li X., Liu C., Sun Y. (2025). Remodeling tumor microenvironment by versatile nanoplatform orchestrated mechanotherapy with chemoimmunotherapy to synergistically enhance anticancer efficiency. Biomaterials.

[148] Han H.S., Choi K.Y. (2021). Advances in nanomaterial-mediated photothermal cancer therapies: toward clinical applications. Biomedicines.

[149] Zhao L., Zhu H., Duo Y.Y., Wang Z.G., Pang D.W., Liu S.L. (2024). A cyanine with 83.2% photothermal conversion efficiency and absorption wavelengths over 1200 nm for photothermal therapy. Adv. Healthcare Mater..

[150] Yang M., Ou X., Li J., Sun J., Zhao Z., Lam J.W.Y. (2024). BF(2)-Bridged azafulvene dimer-based 1064 nm laser-driven superior photothermal agent for deep-seated tumor therapy. Angew Chem. Int. Ed. Engl..

[151] Cui X., Cao C., Hao W., Pan X., Cao Y., Fu Y. (2025). A nanoplatform of reversing tumor immunosuppressive microenvironment based on the NIR-II gold hollow nanorod for the treatment of hepatocellular carcinoma. Small.

[152] Overchuk M., Weersink R.A., Wilson B.C., Zheng G. (2023). Photodynamic and photothermal therapies: synergy opportunities for nanomedicine. ACS Nano.

[153] Yang T., Dai L., Liu J., Lu Y., Pan M., Pan L. (2025). Metal-phenolic-network-coated gold nanoclusters for enhanced photothermal/chemodynamic/immunogenic cancer therapy. Bioact. Mater..

[154] Obaid G., Celli J.P., Broekgaarden M., Bulin A.L., Uusimaa P., Pogue B. (2024). Engineering photodynamics for treatment, priming and imaging. Nat. Rev. Bioeng..

[155] Lin Y., Kong X., Liu Z. (2025). Engineered photoactivatable nanomicelles for ferroptosis-like combinational tumor therapy in vitro and in vivo. ACS Appl. Mater. Interfaces.

[156] Lee D., Kwon S., Jang S.Y., Park E., Lee Y., Koo H. (2022). Overcoming the obstacles of current photodynamic therapy in tumors using nanoparticles. Bioact. Mater..

[157] Li X., Gao J., Wu C., Wang C., Zhang R., He J. (2024). Precise modulation and use of reactive oxygen species for immunotherapy. Sci. Adv..

[158] Wang P., Yu L.B., Shen Q.H., Dao J., Di Z.Y., Li Z.Y. (2025). Synergistic copper-Coordination/Glutathione reduction drives an in situ type II-to-Type I photodynamic switch in iridium-based photosensitizer nanocomposites for potentiated cancer immunotherapy. Adv. Mater..

[159] Liu X., Zhang Y., Wang Y., Zhu W., Li G., Ma X. (2020). Comprehensive understanding of magnetic hyperthermia for improving antitumor therapeutic efficacy. Theranostics.

[160] Shakeri-Zadeh A., Bulte J.W.M. (2024). Imaging-guided precision hyperthermia with magnetic nanoparticles. Nat. Rev. Bioeng..

[161] Lee J.Y., Na Y.R., Na C.M., Im P.W., Park H.W., Kim M.K. (2025). 7-nm Mn(0.5) Zn(0.5)Fe(2)O(4) superparamagnetic iron oxide nanoparticle (SPION): a high-performance theranostic for MRI and hyperthermia applications. Theranostics.

[162] Winkler J., Abisoye-Ogunniyan A., Metcalf K.J., Werb Z. (2020). Concepts of extracellular matrix remodelling in tumour progression and metastasis. Nat. Commun..

[163] Xu F., Huang X., Wang Y., Zhou S. (2020). A size-changeable collagenase-modified nanoscavenger for increasing penetration and retention of nanomedicine in deep tumor tissue. Adv. Mater..

[164] Zhong Y., Zhang J., Zhang J., Hou Y., Chen E., Huang D. (2020). Tumor microenvironment‐activatable nanoenzymes for mechanical remodeling of extracellular matrix and enhanced tumor chemotherapy. Adv. Funct. Mater..

[165] Hu X., Zhang B., Zhang M., Liang W., Hong B., Ma Z. (2024). An artificial metabzyme for tumour-cell-specific metabolic therapy. Nat. Nanotechnol..

[166] Yuan Z., Li Y., Zhang S., Wang X., Dou H., Yu X. (2023). Extracellular matrix remodeling in tumor progression and immune escape: from mechanisms to treatments. Mol. Cancer.

[167] Chen H., Wu F., Xie X., Wang W., Li Q., Tu L. (2021). Hybrid nanoplatform: enabling a precise antitumor strategy via dual-modal imaging-guided Photodynamic/Chemo-/Immunosynergistic therapy. ACS Nano.

[168] Sun P., Hu D., Chen P., Wang X., Shen Q., Chen S. (2024). Anti-quenching NIR-II excitation phenylboronic acid modified conjugated polyelectrolyte for intracellular peroxynitrite-enhanced chemo-photothermal therapy. Adv. Sci. (Weinh.).

[169] Sun R., Ma W., Ling M., Tang C., Zhong M., Dai J. (2022). pH-activated nanoplatform for visualized photodynamic and ferroptosis synergistic therapy of tumors. J. Contr. Release.

[170] Yu Y. (2022). Multi-target combinatory strategy to overcome tumor immune escape. Front. Med..

[171] Chen X., Fan X., Zhang Y., Wei Y., Zheng H., Bao D. (2022). Cooperative coordination-mediated multi-component self-assembly of “all-in-one” nanospike theranostic nano-platform for MRI-guided synergistic therapy against breast cancer. Acta Pharm. Sin. B.

[172] Xing Y., Li J., Wang L., Zhu Z., Yan J., Liu Y. (2025). A bifunctional lysosome-targeting chimera nanoplatform for tumor-selective protein degradation and enhanced cancer immunotherapy. Adv. Mater..

[173] Chen H., Liu J., Cao Z., Li J., Zhang H., Yang Q. (2025). Enhancing hepatocellular carcinoma therapy with DOX-loaded SiO(2) nanoparticles via mTOR-TFEB pathway autophagic flux inhibition. J. Nanobiotechnol..

[174] Yang Y., Liu X., Zhang R., Liu Y., Zhou N., Jiang Y. (2025). Size-tunable micro-nano liposomes: enhanced lung targeting and tumor penetration for combination treatment of lung cancer. Small.

[175] Saha T., Fojtu M., Nagar A.V., Thurakkal L., Srinivasan B.B., Mukherjee M. (2024). Antibody nanoparticle conjugate-based targeted immunotherapy for non-small cell lung cancer. Sci. Adv..

[176] Wang S., Wang H., Drabek A., Smith W.S., Liang F., Huang Z.R. (2024). Unleashing the potential: designing antibody-targeted lipid nanoparticles for industrial applications with CMC considerations and clinical outlook. Mol. Pharm..

[177] Panikar S.S., Banu N., Haramati J., Del Toro-Arreola S., Riera Leal A., Salas P. (2021). Nanobodies as efficient drug-carriers: progress and trends in chemotherapy. J. Contr. Release.

[178] Bekes M., Langley D.R., Crews C.M. (2022). PROTAC targeted protein degraders: the past is prologue. Nat. Rev. Drug Discov..

[179] Liu Z., Hu M., Yang Y., Du C., Zhou H., Liu C. (2022). An overview of PROTACs: a promising drug discovery paradigm. Mol. Biomed..

[180] Chen J., Qiu M., Ma F., Yang L., Glass Z., Xu Q. (2021). Enhanced protein degradation by intracellular delivery of pre-fused PROTACs using lipid-like nanoparticles. J. Contr. Release.

[181] Ma J., Fang L., Sun Z., Li M., Fan T., Xiang G. (2024). Folate-PEG-PROTAC micelles for enhancing tumor-specific targeting proteolysis in vivo. Adv. Healthcare Mater..

[182] Yang L., Yang Y., Zhang J., Li M., Yang L., Wang X. (2024). Sequential responsive nano-PROTACs for precise intracellular delivery and enhanced degradation efficacy in colorectal cancer therapy. Signal Transduct. Targeted Ther..

[183] Wang S.W., Gao C., Zheng Y.M., Yi L., Lu J.C., Huang X.Y. (2022). Current applications and future perspective of CRISPR/Cas9 gene editing in cancer. Mol. Cancer.

[184] Kon E., Ad-El N., Hazan-Halevy I., Stotsky-Oterin L., Peer D. (2023). Targeting cancer with mRNA-lipid nanoparticles: key considerations and future prospects. Nat. Rev. Clin. Oncol..

[185] He X., Li G., Huang L., Shi H., Zhong S., Zhao S. (2025). Nonviral targeted mRNA delivery: principles, progresses, and challenges. MedComm.

[186] Zhang S., Shen J., Li D., Cheng Y. (2021). Strategies in the delivery of Cas9 ribonucleoprotein for CRISPR/Cas9 genome editing. Theranostics.

[187] Wu F., Li N., Xiao Y., Palanki R., Yamagata H., Mitchell M.J. (2025). Lipid nanoparticles for delivery of CRISPR gene editing components. Small Methods.

[188] Qin J., Liu J., Wei Z., Li X., Chen Z., Li J. (2025). Targeted intervention in nerve-cancer crosstalk enhances pancreatic cancer chemotherapy. Nat. Nanotechnol..

[189] Linderman S.W., DeRidder L., Sanjurjo L., Foote M.B., Alonso M.J., Kirtane A.R. (2025). Enhancing immunotherapy with tumour-responsive nanomaterials. Nat. Rev. Clin. Oncol..

[190] Wang Z.H., Zeng X., Huang W., Yang Y., Zhang S., Yang M. (2025). Bioactive nanomotor enabling efficient intestinal barrier penetration for colorectal cancer therapy. Nat. Commun..

[191] Croitoru G.A., Pirvulescu D.C., Niculescu A.G., Epistatu D., Radulescu M., Grumezescu A.M. (2024). Nanomaterials in immunology: bridging innovative approaches in immune modulation, diagnostics, and therapy. J. Funct. Biomater..

[192] Wang X., Zhao J., Marostica E., Yuan W., Jin J., Zhang J. (2024). A pathology foundation model for cancer diagnosis and prognosis prediction. Nature.

[193] Haug C.J., Drazen J.M. (2023). Artificial intelligence and machine learning in clinical medicine, 2023. N. Engl. J. Med..

[194] Bhinder B., Gilvary C., Madhukar N.S., Elemento O. (2021). Artificial intelligence in cancer research and precision medicine. Cancer Discov..

[195] Chang T.G., Park S., Schaffer A.A., Jiang P., Ruppin E. (2025). Hallmarks of artificial intelligence contributions to precision oncology. Nat. Cancer.

[196] Chou W.C., Chen Q., Yuan L., Cheng Y.H., He C., Monteiro-Riviere N.A. (2023). An artificial intelligence-assisted physiologically-based pharmacokinetic model to predict nanoparticle delivery to tumors in mice. J. Contr. Release.

[197] Wang Q., Liu Y., Li C., Xu B., Xu S., Liu B. (2025). Machine learning-enhanced nanoparticle design for precision cancer drug delivery. Adv. Sci. (Weinh.).

[198] Zhang C., Yuan Y., Xia Q., Wang J., Xu K., Gong Z. (2025). Machine learning-driven prediction, preparation, and evaluation of functional nanomedicines via drug-drug self-assembly. Adv. Sci. (Weinh.).

[199] Rafiei M., Shojaei A., Chau Y. (2025). Machine learning-assisted design of immunomodulatory lipid nanoparticles for delivery of mRNA to repolarize hyperactivated microglia. Drug Deliv..

[200] Chan A., Kirtane A.R., Qu Q.R., Huang X., Woo J., Subramanian D.A. (2025). Designing lipid nanoparticles using a transformer-based neural network. Nat. Nanotechnol..

[201] Xu Y., Ma S., Cui H., Chen J., Xu S., Gong F. (2024). AGILE platform: a deep learning powered approach to accelerate LNP development for mRNA delivery. Nat. Commun..

[202] Wang W., Chen K., Jiang T., Wu Y., Wu Z., Ying H. (2024). Artificial intelligence-driven rational design of ionizable lipids for mRNA delivery. Nat. Commun..

[203] Song Q., Li Y., Ma L., Li Y., Lv Y. (2024). A high-throughput screening strategy for synthesizing molecularly imprinted polymer nanoparticles selectively targeting tumors. Adv. Healthcare Mater..

[204] Sammut S.J., Crispin-Ortuzar M., Chin S.F., Provenzano E., Bardwell H.A., Ma W. (2022). Multi-omic machine learning predictor of breast cancer therapy response. Nature.

[205] Ren Y.F., Ma Q., Zeng X., Huang C.X., Ren J.L., Li F. (2024). Single-cell RNA sequencing reveals immune microenvironment niche transitions during the invasive and metastatic processes of ground-glass nodules and part-solid nodules in lung adenocarcinoma. Mol. Cancer.

[206] Tang J., Zhang J., Li Y., Hu Y., He D., Ni H. (2025). Interpretable radiomics model predicts nanomedicine tumor accumulation using routine medical imaging. Adv. Mater..

[207] Zhu L., Hu J., Wu X., Zhang J., Xu X., Huang X. (2025). Programmed enhancement of endogenous iron-mediated lysosomal membrane permeabilization for tumor ferroptosis/pyroptosis dual-induction. Nat. Commun..

[208] Navarro G., Gomez-Autet M., Morales P., Rebassa J.B., Llinas Del Torrent C., Jagerovic N. (2024). Homodimerization of CB(2) cannabinoid receptor triggered by a bivalent ligand enhances cellular signaling. Pharmacol. Res..

[209] Wu J., Ma T., Zhu M., Mu J., Huang T., Xu D. (2024). A pluripotential neutrophil-mimic nanovehicle modulates immune microenvironment with targeted drug delivery for augmented antitumor chemotherapy. ACS Nano.

[210] Chen S.X., Zhang J., Xue F., Liu W., Kuang Y., Gu B. (2023). In situ forming oxygen/ROS-responsive niche-like hydrogel enabling gelation-triggered chemotherapy and inhibition of metastasis. Bioact. Mater..

[211] Li X., Liu H., Gao W., Yang Q., Li X., Zhou X. (2024). Octadecyl gallate and lipid-modified MnSe(2) nanoparticles enhance radiosensitivity in esophageal squamous cell carcinoma and promote radioprotection in normal tissues. Adv. Mater..

[212] Yang W., Feng Z., Lai X., Li J., Cao Z., Jiang F. (2024). Calcium nanoparticles target and activate T cells to enhance anti-tumor function. Nat. Commun..

[213] Jiang Y., Cao H., Deng H., Guan L., Langthasa J., Colburg D.R.C. (2024). Gold-siRNA supraclusters enhance the anti-tumor immune response of stereotactic ablative radiotherapy at primary and metastatic tumors. Nat. Biotechnol..

[214] Mellman I., Chen D.S., Powles T., Turley S.J. (2023). The cancer-immunity cycle: indication, genotype, and immunotype. Immunity.

[215] Huntington N.D., Cursons J., Rautela J. (2020). The cancer-natural killer cell immunity cycle. Nat. Rev. Cancer.

[216] Arner E.N., Rathmell J.C. (2023). Metabolic programming and immune suppression in the tumor microenvironment. Cancer Cell.

[217] Jin Y., Huang Y., Ren H., Huang H., Lai C., Wang W. (2024). Nano-enhanced immunotherapy: targeting the immunosuppressive tumor microenvironment. Biomaterials.

[218] Li X., Ma Y., Xin Y., Ma F., Gao H. (2023). Tumor-targeting nanoassembly for enhanced colorectal cancer therapy by eliminating intratumoral Fusobacterium nucleatum. ACS Appl. Mater. Interfaces.

[219] Jiang J., Zheng H., Wang Z., Wang X., Xie Q., Liu X. (2025). Intracellular dehydrogenation catalysis leads to reductive stress and immunosuppression. Nat. Nanotechnol..

[220] Finbloom J.A., Huynh C., Huang X., Desai T.A. (2023). Bioinspired nanotopographical design of drug delivery systems. Nat. Rev. Bioeng..

[221] Tavakkoli Yaraki M., Liu B., Tan Y.N. (2022). Emerging strategies in enhancing singlet oxygen generation of nano-photosensitizers toward advanced phototherapy. Nano-Micro Lett..

[222] Zhang T., Li J., Lu J., Li J., Zhang H., Miao Y. (2025). Enhanced tumor-targeting ability of transferrin-functionalized magnetic nanoparticles by in vivo AMF stimulation. Biomaterials.

[223] Wang Q., He J., Qi Y., Ye Y., Ye J., Zhou M. (2024). Ultrasound-enhanced nano catalyst with ferroptosis-apoptosis combined anticancer strategy for metastatic uveal melanoma. Biomaterials.

[224] Liu J., Liu Y., Zhi S., Yang Y., Kim H., Wu D. (2025). A nanotherapeutic agent for synergistic tumor therapy: co-activation of photochemical-biological effects. Angew Chem. Int. Ed. Engl..

[225] Qian X., Yi W., Yan W., Cai Y., Hu S., Yan D. (2025). Cryo-shocked tumor-reprogrammed sonosensitive antigen-presenting cells improving sonoimmunotherapy via T cells and NK cells immunity. Adv. Mater..

[226] Wang J., Zhao S., Yi J., Sun Y., Agrawal M., Oelze M.L. (2024). Injectable mechanophore nanoparticles for deep-tissue mechanochemical dynamic therapy. ACS Nano.

[227] Cao Y., Zhong X., Wu N., Wan L., Tang R., He H. (2024). An ultrasound‐responsive and in situ gelling hydrogel nanocomposite for boosting anti PD‐L1 immunotherapy via remodeling aberrant ECM of post‐surgical residual cancer. Adv. Funct. Mater..

[228] Zhang S., Wang X., Chen X., Shu D., Lin Q., Zou H. (2025). An on-Demand oxygen nano-vehicle sensitizing protein and nucleic acid drug augment immunotherapy. Adv. Mater..

[229] Wei R., Xie K., Li T., Lin W., Zhao Y., Li J. (2025). Immunity/Metabolism dual-regulation via an acidity-triggered bioorthogonal assembly nanoplatform enhances glioblastoma immunotherapy by targeting CXCL12/CXCR4 and adenosine-A2AR pathways. Biomaterials.

[230] Vincent M.P., Navidzadeh J.O., Bobbala S., Scott E.A. (2022). Leveraging self-assembled nanobiomaterials for improved cancer immunotherapy. Cancer Cell.

[231] Sun J., Jiang K., Wang Y., Liu Y., Wang T., Ding S. (2023). One-pot synthesis of tumor-microenvironment responsive degradable nanoflower-medicine for multimodal cancer therapy with reinvigorating antitumor immunity. Adv. Healthcare Mater..

[232] Yang Y., Liu Q., Wang M., Li L., Yu Y., Pan M. (2024). Genetically programmable cell membrane-camouflaged nanoparticles for targeted combination therapy of colorectal cancer. Signal Transduct. Targeted Ther..

[233] Zhang H., Ren Y., Hou L., Chang J., Zhang Z., Zhang H. (2020). Positioning remodeling nanogels mediated codelivery of antivascular drug and autophagy inhibitor for cooperative tumor therapy. ACS Appl. Mater. Interfaces.

[234] Abrishami A., Bahrami A.R., Nekooei S., Sh Saljooghi A., Matin M.M. (2024). Hybridized quantum dot, silica, and gold nanoparticles for targeted chemo-radiotherapy in colorectal cancer theranostics. Commun. Biol..

[235] Rezaei B., Yari P., Sanders S.M., Wang H., Chugh V.K., Liang S. (2024). Magnetic nanoparticles: a review on synthesis, characterization, functionalization, and biomedical applications. Small.

[236] Jiang J., Cui X., Huang Y., Yan D., Wang B., Yang Z. (2024). Advances and prospects in integrated nano-oncology. Nano Biomed. Eng..

[237] Wu H., Wang Y., Ren Z., Cong H., Shen Y., Yu B. (2025). The nanocarrier strategy for crossing the blood-brain barrier in glioma therapy. Chin. Chem. Lett..

[238] Liu S., Ren Z., Yan M., Ye W., Hu Y. (2025). Strategies to enhance the penetration of nanomedicine in solid tumors. Biomaterials.

[239] Cao J., Huang D., Peppas N.A. (2020). Advanced engineered nanoparticulate platforms to address key biological barriers for delivering chemotherapeutic agents to target sites. Adv. Drug Deliv. Rev..

[240] Khan S., Falahati M., Cho W.C., Vahdani Y., Siddique R., Sharifi M. (2023). Core-shell inorganic NP@MOF nanostructures for targeted drug delivery and multimodal imaging-guided combination tumor treatment. Adv. Colloid Interface Sci..

[241] Zhang J., Li W., Qi Y., Wang G., Li L., Jin Z. (2023). PD-L1 aptamer-functionalized metal-organic framework nanoparticles for robust photo-immunotherapy against cancer with enhanced safety. Angew Chem. Int. Ed. Engl..

[242] Du J., Jia T., Li F., Li Y., Wang Q., He L. (2024). MOF‐Coated upconversion nanoparticle agents enable synergistic photodynamic therapy and immunotherapy. Adv. Funct. Mater..

[243] Deng W., Wang Y., Wang J., Su Y., Li M., Qu K. (2025). Leveraging vitamin C to augment nanoenabled photothermal immunotherapy. ACS Nano.

[244] Xing W., Li T., Yang G., Wu S., Pang B., Xu Y. (2025). Thermo-responsive gold nanorod vesicles for combined NIR-II photothermal therapy and chemotherapy of solid tumors. Acta Biomater..

[245] Yan D., Wang M., Wu Q., Niu N., Li M., Song R. (2022). Multimodal imaging-guided photothermal immunotherapy based on a versatile NIR-II aggregation-induced emission luminogen. Angew Chem. Int. Ed. Engl..

[246] Chai Y., Xu M., Sun Y., Zhu Y., Du T., Zhu B. (2025). Hydrogen sulfide-responsive and depleting NIR-II nanoplatform synergistic photodynamic therapy for colorectal cancer. Chem. Eng. J..

[247] Ke L., Wei F., Xie L., Karges J., Chen Y., Ji L. (2022). A biodegradable Iridium(III) coordination polymer for enhanced two-photon photodynamic therapy using an apoptosis-ferroptosis hybrid pathway. Angew Chem. Int. Ed. Engl..

[248] Gao M., Sun Q., Zhang H., Liu M., Peng R., Qin W. (2024). Bioinspired nano-photosensitizer-activated Caspase-3/GSDME pathway induces pyroptosis in lung cancer cells. Adv. Healthcare Mater..

[249] Lu Y., Huang C., Fu W., Gao L., Mi N., Ma H. (2024). Design of the distribution of iron oxide (Fe(3)O(4)) nano-particle drug in realistic cholangiocarcinoma model and the simulation of temperature increase during magnetic induction hyperthermia. Pharmacol. Res..

